# Heterogeneity and longitudinal transcriptomic characteristics of Tregs in COVID-19 patients

**DOI:** 10.3389/fimmu.2025.1548173

**Published:** 2025-03-06

**Authors:** Yanling Wen, Juanjuan Zhao, Zheng Zhang

**Affiliations:** Institute for Hepatology, National Clinical Research Center for Infectious Disease, Shenzhen Third People’s Hospital, The Second Affiliated Hospital, School of Medicine, Southern University of Science and Technology, Shenzhen, Guangdong, China

**Keywords:** Tregs, scRNA-seq, COVID-19, heterogeneity, dynamic change

## Abstract

**Introduction:**

Regulatory T cells (Tregs) play a crucial role in maintaining immune tolerance by suppressing immune responses against pathogens. The fluctuation of Treg proportions in COVID-19 remains a topic of debate, and the mechanisms triggering Treg activation in COVID-19 are still unclear. Understanding these issues is essential for better managing immune responses in COVID-19 patients.

**Methods:**

We collected a cohort of COVID-19 patients with varying disease severity and stage to explore the transcriptomic and functional traits of Tregs in these individuals. Using transcriptomic analysis, we evaluated the proportion and functionality of different Treg subsets, specifically HLA_DR^+^ Tregs, across different stages of COVID-19 patients.

**Results:**

Our analysis revealed that the proportion of *CCR7* ^+^ Tregs decreased as the disease advanced, while the cell proportion of HLA_DR^+^ regs escalated with the severity of the disease. Moreover, the transcription actor *CARHSP1* exhibited apositive correlation with the proportion of HLA_DR^+^ Tregs. Notably, the heightened suppressive function of HLA_DR^+^ Tregs in severe COVID-19 patients, with interactions between *PF4* and *CXCR3*, contributed to the homeostasis of HLA_DR^+^ Tregs in severe COVID-19 patients. Furthermore, we observed that Tregs in COVID-19 patients exhibited weakened TCR clonotype expansion, and the suppression of HLA_DR^+^ Tregs with expanded TCR clonotypes in severe COVID-19 cases did not show a significant increase compared to asymptomatic and mild COVID-19 groups. The findings indicate that Tregs may be activated through the bystander effect, as evidenced by the analysis of TCR clonotype characteristics.

**Discussion:**

Our research delineates the diversity of dynamic alterations in Tregs and sheds light on potential mechanisms underlying Treg activation, providing a theoretical foundation and offering treatment strategies for managing COVID-19 patients.

## Introduction

Regulatory T cells (Tregs) are a subset of CD4^+^ T cells with high expression of CD25 and FOXP3 (1). Tregs play a key role in maintaining immune tolerance, which can prevent the onset of autoimmune diseases (2) and inhibit the anti-tumor or anti-pathogen immune responses (3). Tregs suppress immune responses through several mechanisms (4): (1) secretion of immunosuppressive cytokines (TGF-β, IL-10, and IL-35) (5, 6); (2) induction of effector cell apoptosis via granzyme, and perforin; (3) disruption of immune cell metabolism through IL-2 receptor, cAMP inhibition, and A2 adenosine receptor modulation; and (4) interaction with dendritic cells to alter their function and maturation. Tregs are mainly divided into two types: natural Tregs (nTregs) (7), which develop in the thymus and are involved in providing tolerance to autoantigens (8), and induced Tregs (iTregs), which are transformed from naïve CD4^+^ T cells, can be expressed *in vivo* and *in vitro* (9), and are mainly involved in preventing local inflammation in the presence of exogenous antigens.

As of 6 October 2024, SARS-CoV-2 has resulted in the death of at least 7.07 million people (https://data.who.int/). Although the current COVID-19 pandemic no longer constitutes a “Public Health Emergency of International Concern,” SARS-CoV-2 infection is still a serious threat to human health, especially to the elderly and to patients with low immunity and other high-risk groups who are infected are still likely to develop into a severe life-threatening illness. A number of studies have revealed that immunological perturbations are associated with COVID-19 severity, such as increased immature myeloid suppressor cells (10, 11), T cells lymphopenia (12), and cytokine storm (13, 14). The immune system releases a large number of pro-inflammatory cytokines in response to SARS-CoV-2 invasion. However, uncontrolled inflammation can damage tissues such as the lungs, heart, liver, and kidneys, potentially leading to respiratory failure or multiple organ failure (15). During the SARS-CoV-2 infection process, Tregs may play a dual role. On the one hand, Tregs play a crucial role in regulating the immune response in COVID-19, preventing cytokine storms and tissue damage. On the other hand, Tregs may also inhibit innate and adaptive antiviral immune responses.

Currently, there is still controversy in the research community regarding the variation in the proportion of Tregs in COVID-19 patients, primarily manifesting in two aspects. On the one hand, there is the change in the proportion of Tregs in COVID-19 patients compared to healthy individuals, and on the other hand, there is the change in the proportion of Tregs in severe COVID-19 patients compared to mild cases. Compared to healthy individuals, some studies have found that the proportion of Tregs in patients with COVID-19 does not show a significant difference (16–18). However, some studies have suggested that the proportion of Tregs was elevated in patients with COVID-19 (19–23), while others indicate that the proportion of Tregs in COVID-19 patients was decreased (24, 25). Meanwhile, compared to mild patients, some studies have suggested that there was no significant difference in the proportion of Tregs in severe cases (16–19, 26). However, some studies have indicated a significant increase in the proportion of Tregs in severe cases (21, 23), while others showed a significant decrease in the proportion of Tregs in severe patients when compared to mild patients (25, 27, 28). Some researchers have suggested possible reasons for these different results, for instance, the use of non-uniform Treg markers in different studies, varying immune responses induced by infection with different variants of SARS-CoV-2, the lack of standardized criteria to define disease severity and Treg subtypes, patients being at different stages of the disease (29), and the age of the patients. However, these speculations are not supported by evidence.

Some studies have noted that Tregs are highly activated in patients with critical COVID-19 and heightened suppression (30, 31). Hypoxia and high levels of lactic acid have been reported to promote the function of Tregs in COVID-19 (32, 33). Galván-Peña et al. proposed that IL-6 and IL-18 induce the Tregs phenotype in severe COVID-19 patients *in vitro* (30), which has some limitations. TNF/TNFR2 has been recently recognized to play an important role in the function and survival of Tregs. However, the high expression of TNFR2 in COVID-19 patients has not been reported. Therefore, the factors inducing Tregs in COVID-19 remained unclear.

To address these issues, we conducted a longitudinal survey of COVID-19 patients with various outcome categories, which include asymptomatic, mild, and severe cases. Through the utilization of droplet-based single-cell RNA sequencing (scRNA-seq) technology, we constructed a comprehensive dynamic atlas of Tregs from COVID-19 patients. This extensive analysis has yielded invaluable insights into the controversy in research regarding the variation in the proportion of Tregs and the activation mechanism of Tregs in COVID-19 patients. These discoveries not only deepen our understanding of these biological processes but also lay a foundation for theoretical insights and therapeutic approaches in managing COVID-19 patients.

## Results

### Expansion of HLA_DR^+^ Tregs in COVID-19 patients

**Figure 1:**
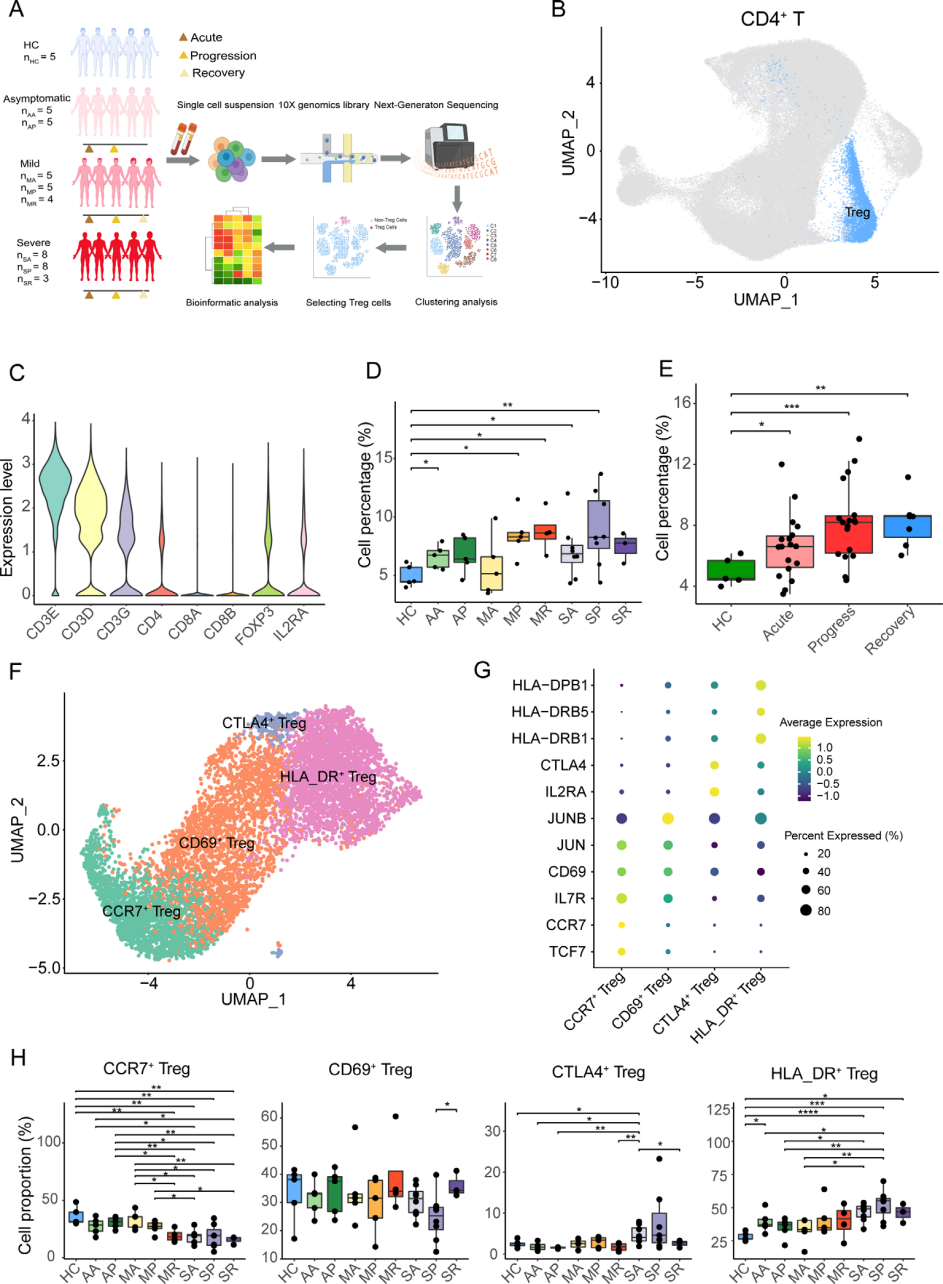
Tregs overrepresentation and Tregs subtypes in COVID-19 patients. **(A)** Overview of the experimental design. Tregs from PBMC samples of asymptomatic, mild, and severe COVID-19 patients, at acute, progressive, and recovery stages, and healthy controls were assessed using single-cell RNA-seq. The groups included: HC (healthy controls), AA (asymptomatic acute), AR (asymptomatic recovery), MA (mild acute), MP (mild progression), MR (mild recovery), SA (severe acute), SP (severe progression), and SR (severe recovery). **(B)** The UMAP plot displays conventional T cells and Tregs from the integrated CD4^+^ T cells. **(C)** The violin plot shows the expression level of specific genes in the Tregs. **(D)** Cell percentage of Tregs among CD4^+^ T cells in eight COVID-19 patient groups and healthy controls. **(E)** Cell percentage of Tregs among CD4^+^ T cells in COVID-19 patients at acute, progression, recovery stages, and healthy controls. **(F)** The UMAP plot displays four subclusters of Tregs, including *CCR7* ^+^ Treg, *CD69* ^+^ Treg, HLA_DR^+^ Treg, and *CTLA4* ^+^ Treg. **(G)** The heatmap plot shows the expression of cell-specific markers for four clusters of Tregs. **(H)** Cell percentages of Treg subclusters among total Tregs in eight COVID-19 patient groups and healthy controls. *P-values* for pairwise comparisons were calculated by unpaired two-tailed Student’s t-test, *P < 0.05, **P < 0.01, ***P < 0.001, ****P < 0.0001.

We downloaded scRNA-seq data from 48 PBMC samples from Gang Xu et al. (34) which included five asymptomatic, five mild, and eight severe COVID-19 patients and five healthy controls (**Figure 1A**). PBMC samples from severe COVID-19 patients were sampled before, during, and after the exacerbation of the disease. Samples were collected approximately 1 week after the onset of symptoms in the severe acute phase (SA), approximately 17 days after the onset of symptoms in the severe progression phase (SP), and approximately 1 month after discharge from the hospital in the severe recovery phase (SR). At matched time points to severe COVID-19, PBMC samples were also collected from mild patients, comprising the acute (MA), progressive (MP), and recovery (MR) phases of mild disease. Additionally, PBMC samples from asymptomatic COVID-19 cases were collected shortly after admission and 1 week later, representing the acute (AA) and recovery (AR) phases of asymptomatic infection, respectively (**Table 1**). The WHO grading scale (WOS) was employed to classify the severity of COVID-19, categorizing patients into eight distinct groups based on varying levels of disease severity. Asymptomatic patients received scores of 0-2, while those with mild COVID-19 were scored between 0-4. Severe patients were classified with scores of 3-5 in SA, 5-7 in SP, and 0 in SR. Detailed clinical information for all samples is listed in **Supplementary Table S1**.

**Table 1:** The healthy controls (n = 5) and COVID-19 patients (n = 18) enrolled in this study.

Sample NO.	Severity	Stages		
		Acute	Progress	Recovery
S1	Severe	Y	Y	
S2		Y	Y	Y
S3		Y	Y	
S4		Y	Y	Y
S5		Y	Y	Y
S6		Y	Y	
S7		Y	Y	
S8		Y	Y	
M1	Mild	Y	Y	Y
M2		Y	Y	
M3		Y	Y	Y
M4		Y	Y	Y
M5		Y	Y	Y
A1	Asymptomatic	Y		Y
A2		Y		Y
A3		Y		Y
A4		Y		Y
A5		Y		Y
H2	Healthy Contorls			
H3				
H4				
H5				
H6				

Y, sampled; blank, not sampled.

The scRNA-seq datasets were analyzed using the Seurat program, and CD4^+^ T cells were isolated by *CD3E*, *CD3D*, *CD3G*, *CD8A*, *CD8B*, and *CD4* marker genes (**Figure 1B**, **Supplementary Figures S1A, B**). CD4^+^ T cells were further re-clustered, and Tregs were sorted out by *CD3E, CD3D, CD3G, CD8A, CD8B, CD4, FOXP3*, and *IL2RA* genes (**Figure 1C**, **Supplementary Figures S1C, D**). We found that the percentage of Tregs to CD4^+^ T cells in COVID-19 patients was higher than that in healthy controls, especially in the AA, MP, MR, SA, and SP groups (**Figure 1D**). Meanwhile, compared with healthy controls at different stages, such as the acute phase, progressive phase, and convalescent phase, the proportion of Tregs in CD4^+^ T cells increased in COVID-19 patients, especially in the acute stage and progressive stages (**Figure 1E**).

The sorted Tregs were re-clustered into four clusters and annotated by marker genes, namely, *CCR7* ^+^ Tregs (expressing *TCF7*, *CCR7*, and *IL7R*), *CD69* ^+^ Tregs (expressing *CD69*, *JUN*, and *JUNB*), *CTLA4* ^+^ Tregs (expressing *IL2RA* and *CTLA4*), and HLA_DR^+^ Tregs (expressing *HLA_DRB1*, *HLA_DRB5*, and *HLA-DPB1*) (**Figures 1F, G**). Except for the AR group, the proportion of *CCR7* ^+^ Tregs in the other eight groups was lower in the healthy controls, especially in the MR, SA, SP, and SR groups (**Figure 1G**). Compared with the SP group, *CD69* ^+^ Tregs were significantly increased in the SR group. Compared with healthy controls, the percentage of *CD69* ^+^ Tregs in the eight groups did not change significantly (**Figure 1H**). Except for the SA group, the proportion of *CTLA4* ^+^ Tregs in the other seven groups showed no significant changes compared to healthy controls (**Figure 1H**). Interestingly, the average proportion of HLA_DR^+^ Tregs in the eight COVID-19 patient groups was higher than that in the healthy controls, with the highest proportion observed in the severe progression group (**Figure 1H**).

Collectively, except for the MA group, the proportion of Tregs in COVID-19 patients was slightly higher compared to healthy individuals. After subdividing Tregs into four subclusters (*CCR7* ^+^ Tregs, *CD69* ^+^ Tregs, *CTLA4* ^+^ Tregs, and HLA_DR^+^ Tregs), the percentage of *CCR7* ^+^ Tregs in the eight COVID-19 patient groups was lower compared to the healthy controls, while the proportion of HLA_DR^+^ Tregs in the eight COVID-19 patient groups was higher.

### Positive correlation between the expression of transcription factor *CARHSP1* and proportion of HLA_DR^+^ Tregs

**Figure 2:**
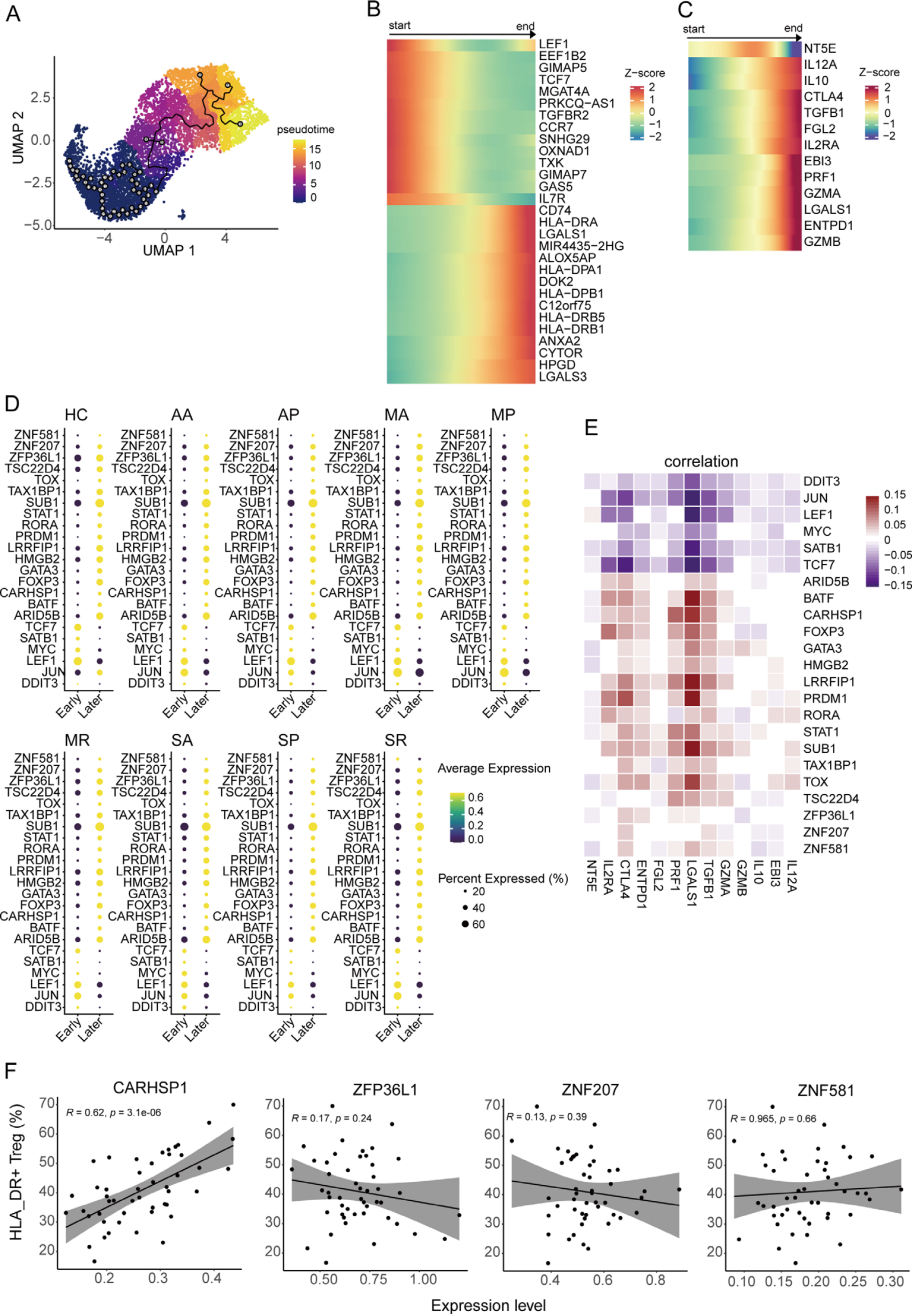
Pseudo-temporal trajectory of Tregs. **(A)** The UMAP plot shows the pseudo-temporal developmental trajectory of Tregs through monocle3. **(B, C)** The heatmap plots display the dynamic expression pattern of the top 20 highly expressed genes during the early and late periods along with the pseudo-temporal trajectory of Tregs **(B)** and genes associated with suppressive function along with the pseudo-temporal trajectory of Tregs **(C)**. **(D)** The bubble heatmap plot shows the expression level of the differentially expressed transcription factors (TFs) between early periods and late periods from eight COVID-19 patient groups and healthy controls. Colors indicate the expression level, while the size of the circles represents the proportion of expressed cells. **(E)** Correlograms visualize the correlation between expression level of TFs and suppression genes in COVID-19 patients. **(F)** The correlation between the gene expression level of four TFs and the cell percentage of HLA-DR^+^ Tregs, including *CARHSP1*, *ZFP36L1*, *ZNF581*, and *ZNF207*.

Through single-cell trajectory analysis using Monocle3, we found that the differentiation and developmental trajectory of the four subclusters of Tregs were in the following order: *CCR7* ^+^ Treg-> *CD69* ^+^ Treg-> *CTLA4* ^+^ Treg-> HLA_DR^+^ Treg (**Figure 2A**, **Supplementary Figures S2A–D**). The top 20 genes highly expressed in early-stage Tregs mainly included *LEF1*, *TCF7, CCR7*, *MYC*, and *IL7R*, while the top 20 genes highly expressed in late-stage Tregs were mainly *HLA_DRA*, *HLA-DPA1*, *HLA_DRB5*, and *HLA_DRB1* (**Figure 2B**). In addition, most suppressive genes were highly expressed in the final stages of Treg development, with only *NT5E* in the middle stages (**Figure 2C**). The above showed that HLA_DR^+^ Tregs at the end of differentiation were a group of mature Tregs accompanied by a high expression of suppressive genes.

In order to discover the key transcription factors that regulate Treg development, we compared the differentially expressed genes between early- and late-developing Tregs from each group, overlaid the differentially expressed genes and transcription factor database downloaded from AnimalTFDB (version 3.0) (35), and found 23 transcription factors involved in Treg development, including four new transcription factors (*ZNF581, ZNF207, ZFP36L1, and CARHSP1*) that have not been reported thus far (**Figure 2D**). The expression of the four transcription factors was notably elevated in Tregs at later stages and exhibited a positive correlation with the expression of suppressive genes. Their expression profiles were more similar to those of *BATF*, *FOXP3*, *STAT1*, and *TOX* (**Figure 2E**). Moreover, we found that the expression of *CARHSP1* was positively correlated with the proportion of HLA_DR^+^ Tregs (R=0.62, *P-value*<0.001), while the expression of the other three genes did not correlate with the ratio of HLA_DR^+^ Tregs (*P-value >*0.05) (**Figure 2F**).

In addition, CARHSP1 can interact with CD74, which supports the accumulation and function of regulatory T cells (36) (**Supplementary Figure S2E**). The genes related to HLA_DR were involved in the network, indicating that the downstream genes of CD74 may also regulate the generation of the genes associated with HLA_DR (**Supplementary Figure S2E**).

To summarize, the *CARHSP1* gene potentially regulates the generation of HLA_DR^+^ Tregs, a group of terminally differentiated Tregs.

### Enhanced suppressive function of HLA_DR^+^ Tregs in severe COVID-19 patients

**Figure 3:**
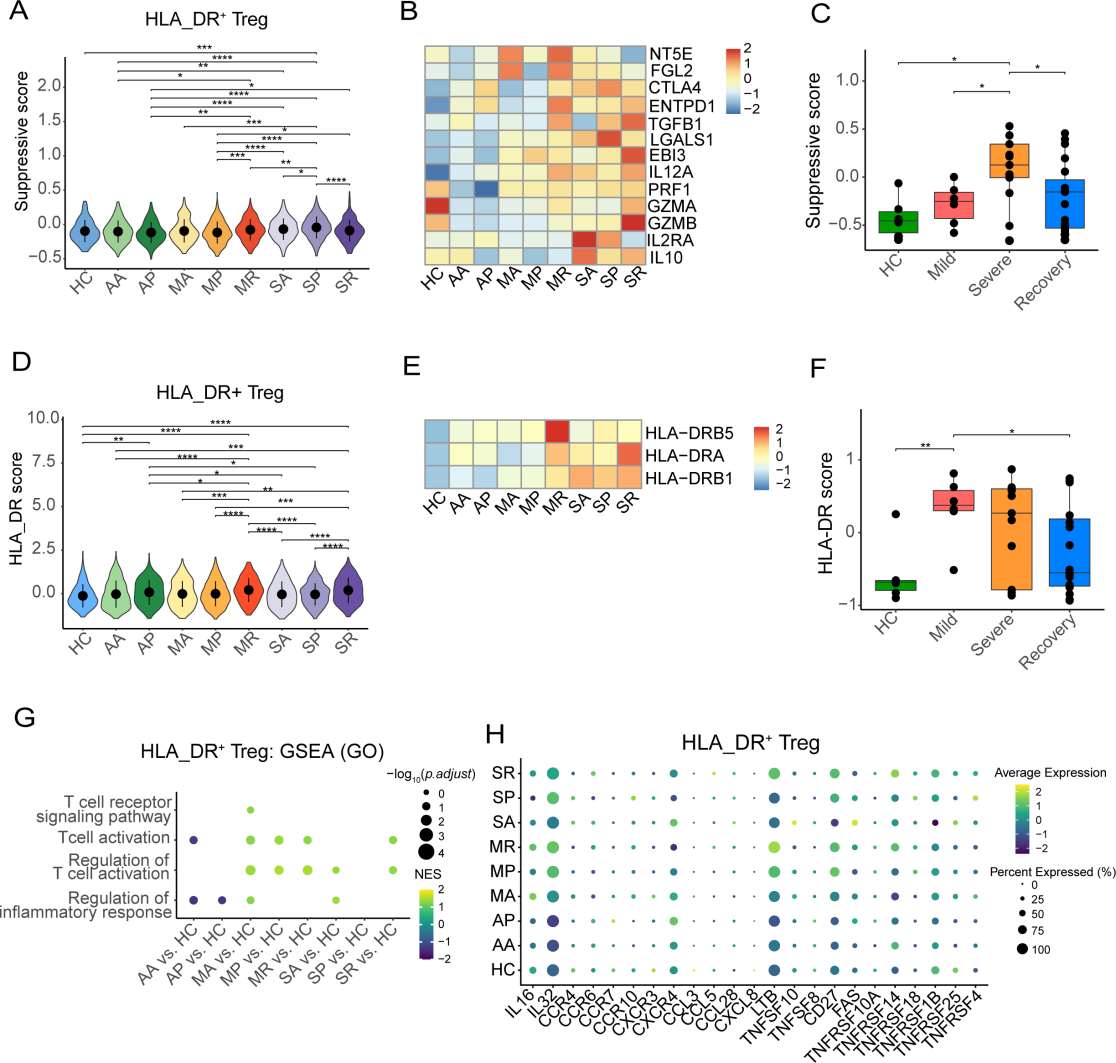
Immune characteristics of HLA-DR^+^ Tregs from HCs and eight COVID-19 patient groups. **(A)** The violin plot shows the suppressive score of eight COVID-19 patient groups and healthy controls in the HLA_DR^+^ Tregs. **(B)** Heatmaps plot displays the expression distribution of the suppression gene of eight COVID-19 patient groups and healthy controls in the HLA_DR^+^ Tregs. **(C)** Box plot shows the distribution of the suppressive score in Tregs across mild, severe, recovery, and HCs by bulk RNA-seq. **(D)** The violin plot shows the HLA-DR score of eight COVID-19 patient groups and healthy controls in the HLA_DR^+^ Tregs. **(E)** Heatmaps plot displays expression distribution of genes associated with HLA_DR score of eight COVID-19 patient group and healthy controls in the HLA_DR^+^ Tregs. **(F)** Box plots show the distribution of HLA-DR score in Tregs across mild, severe, recovery, and HCs by bulk RNA-seq. **(G)** GSEA analysis of the pathway related to T cell activation in the HLA-DR^+^ Tregs. **(H)** The bubble heatmap plot shows the expression level of selected genes associated with cytokine, chemokine receptor, chemokine ligand, TNF family ligand, and TNF family receptor in the HLA-DR^+^ Tregs. *P-values* for pairwise comparisons were calculated by unpaired two-tailed Student’s t-test, *P < 0.05, **P < 0.01, ***P < 0.001, ****P < 0.0001.

Pseudo-temporal analysis indicated that HLA_DR^+^ Tregs are a group of terminally differentiated cells with strong suppressive functions. However, the characteristics of HLA_DR^+^ Tregs in COVID-19 patients of different severity remain unknown. Therefore, by comparing the suppression of HLA_DR^+^ Tregs among HCs and eight groups of COVID-19 patients, we found that HLA_DR^+^ Tregs from the SP group possessed the highest suppressive score compared to the other groups and that in the three stages of severe COVID-19 patients, it was slightly higher than in other COVID-19 patients (**Figure 3A**). Notably, the suppressive function of HLA_DR^+^ Tregs in asymptomatic patients and mild patients showed no difference compared to healthy individuals (**Figure 3A**). In addition, the SP group had a high suppressive score of all Tregs, *CCR7* ^+^ Tregs, *CD69* ^+^ Tregs, and *CTLA4* ^+^ Tregs in severe COVID-19 patients (**Supplementary Figures S3A–D**). Meanwhile, *CTLA4*, *ENTPD1*, *TGFB1*, *LGALS1*, *EBI3*, *IL12A*, *PRF1*, *GZMA*, *GZMB*, *IL2RA*, and *IL10* were highly expressed in severe COVID-19 patients (**Figure 3B**). In addition, compared with healthy subjects and mild and in recovery COVID-19 patients, severe COVID-19 patients exhibited the highest suppressive score, which was calculated from the downloaded bulk RNA-seq data (**Figure 3C**).

Furthermore, we observed that COVID-19 patients exhibited slightly higher HLA_DR scores compared to the healthy controls; the MR group had the highest HLA_DR score among the mild COVID-19 patients, and the SR group had the highest HLA_DR score among the severe COVID-19 patients (**Figure 3D**). However, there was no significant difference between the MR and SR groups regarding the HLA_DR score (**Figure 3D**). *HLA_DRB5*, *HLA_DRB1*, and *HLA_DRA* were expressed at higher levels in COVID-19 patients compared to the healthy controls (**Figure 3E**). Additionally, MR and severe COVID-19 patients showed higher expression levels of *HLA_DRB5*, *HLA_DRB1*, and *HLA_DRA* relative to other COVID-19 patients (**Figure 3E**). Furthermore, based on the analysis of the downloaded bulk transcriptomic data, COVID-19 patients exhibited higher HLA_DR scores compared to healthy individuals. Mild COVID-19 patients had the highest HLA_DR score among the COVID-19 patients. Meanwhile, *HLA_DRB1* and *HLA_DRB5* were significantly highly expressed in the mild group compared to the healthy controls (**Figure 3F**) but not in the severe and recovery groups (**Supplementary Figures S3I, J**).

Furthermore, compared with healthy controls, upregulated genes of HLA_DR^+^ Tregs from the MA, MP, MR, SA, and SR groups were enriched in the T cell activation and regulation of the T cell activation pathway, while the AA group of HLA_DR^+^ Tregs was downregulated in T cell activation pathway and regulation of inflammatory response pathway (**Figure 3G**). Meanwhile, we also detected that the HLA_DR^+^ Tregs in the MA, MP, MR, SA, SP, and SR groups highly expressed IL-32, which is a kind of pro-inflammatory cytokine in humans and encoded by the IL32 gene. *CD27*, *TNFRSF14*, *TNFRS18*, and *TNFRSF4* were highly expressed in the HLA_DR^+^ Tregs from severe COVID-19 patients (**Figure 3H**). However, these genes were not highly expressed in other Treg subsets from the severe COVID-19 patients (**Supplementary Figures S4A–D**).

Taken together, compared to healthy individuals, severe COVID-19 patients demonstrated stronger suppressive capabilities.

### Enhanced interaction between *PF4* of CD14^+^ monocytes and *CXCR3* of HLA_DR^+^ Tregs in severe COVID-19 patients

The effector program of T cells and their expression of immunoregulatory molecules are closely linked to the function of APCs, including dendritic cells (DCs), macrophages, and other monocyte-derived cells. Tumor necrosis factor α (TNFα) promotes iTreg differentiation and function via the TNFR2 signaling pathway, implying that TNFR2 is a critical immune regulator (37). In addition, TNFα is mainly secreted by macrophages and monocytes, and other cells, such as some subsets of T cells, NK cells, B cells, DCs, cardiomyocytes, fibroblasts, and astrocytes, also generate it at low levels (38, 39). Therefore, we took a closer look at what triggers HLA_DR^+^ Treg activation in the myeloid cells of COVID-19 patients.

**Figure 4:**
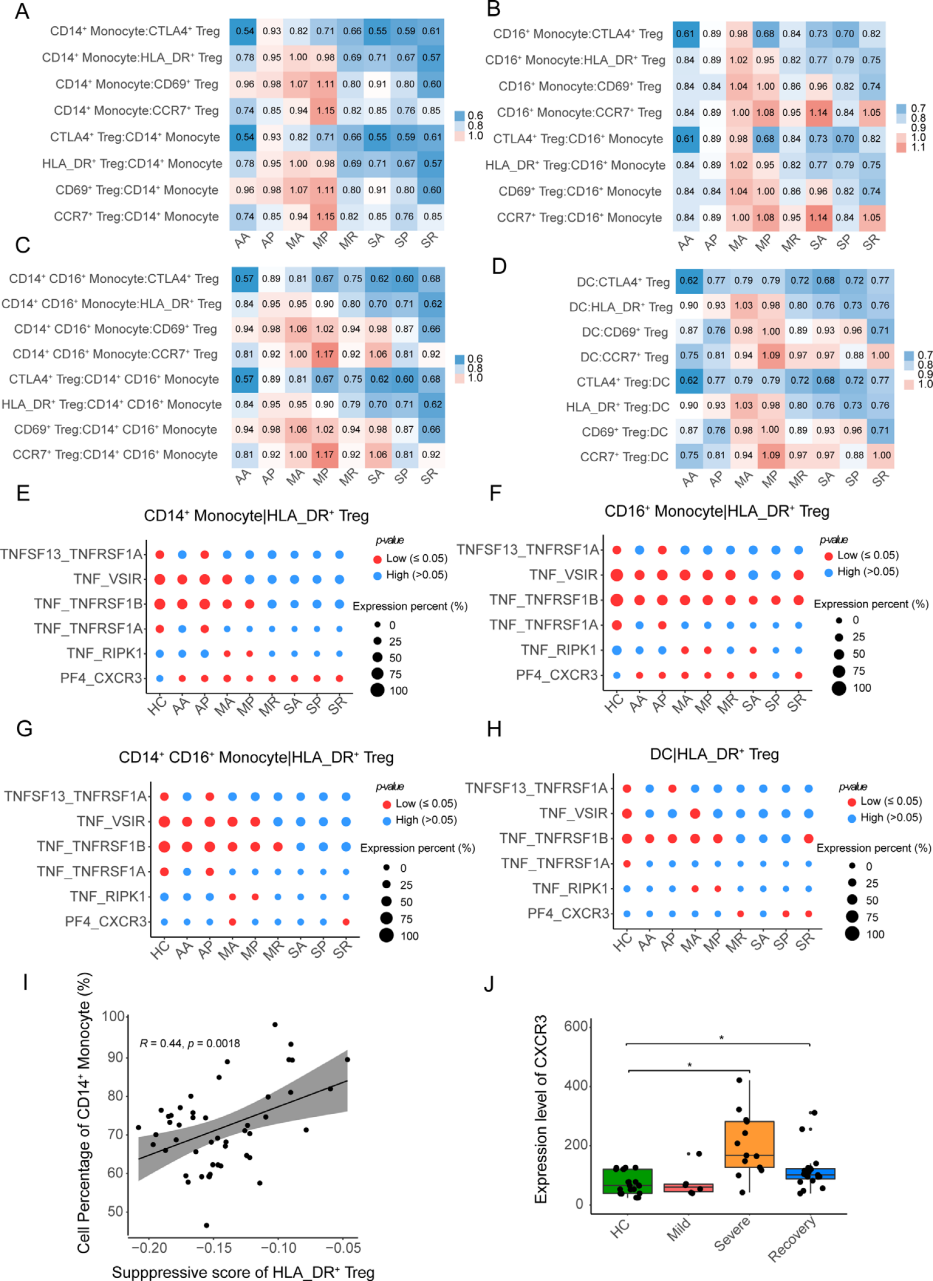
The interactions between APCs and four Treg subclusters. **(A–D)** The heatmap plots show the intensity of the interaction between the four Treg subclusters and CD14^+^ monocytes, CD14^+^ CD16^+^ monocytes, CD16^+^ monocytes, and DCs, respectively. **(E–G)** The bubble heatmap plots show selected L-R pairs between HLA-DR^+^ Tregs and CD14^+^ monocytes, CD14^+^ CD16^+^ monocytes, CD16^+^ monocytes and DCs, respectively. The red color indicates a *P-value* less than 0.05, while the blue color represents a *P-value* more than 0.05. The size of the circles represents the average expression level of ligands and receptors. **(I)** The correlation between the suppressive score of HLA-DR^+^ Tregs and the cell percentage of CD14^+^ monocytes in myeloid cells. **(J)** Box plot shows the expression level of *CXCR3* among HC, mild, severe and recovery COVID-19, the DESseq2 normalized counts as the expression level of *CXCR3*. *P-values* for pairwise comparisons were calculated by unpaired two-tailed Student’s t-test, *P < 0.05.

We explored the interaction of Tregs with other immune cells such as B cells, T cells, myeloid cells, and NK cells (**Supplementary Figures S5A, B**, **Supplementary Table S2**) using the CellphoneDB program and found the interactions between *CD14* ^+^ monocytes and HLA_DR^+^ Tregs were weaker in the SA, SP, and SR groups in severe patients than in the asymptomatic patients and mild patients, and noted that the MA and MP groups had stronger interactions between CD14^+^ monocytes and HLA_DR^+^ Tregs (**Figure 4A**, **Supplementary Table S3**). We also observed the same phenomenon in other myeloid cells, such as CD14^+^ CD16^+^ monocyte cells, CD16^+^ monocyte cells, and DCs (**Figures 4B–D**, **Supplementary Table S3**). As a whole, the interactions between myeloid cells and HLA_DR^+^ Tregs were reduced.

We further investigated the ligands and receptors involved in the interaction between myeloid cells and HLA_DR^+^ Tregs. In the interactions between CD14^+^ monocytes and HLA_DR^+^ Tregs, ligand-receptor pairs including TNF_VSIR, TNF_TNFRSF1B, TNF_TNFRSF1A, and TNF_RIPK1 were weakened in the SA, SP, and SR groups (**Figure 4E**), while the ligand-receptor pair of PF4_CXCR3 was enhanced in the eight COVID-19 patient groups, except for the healthy controls (**Figure 4E**). We also observed that ligand-receptor pairs, including TNF_VSIR, TNF_TNFRSF1B, TNF_TNFRSF1A, and TNF_RIPK1, were weakened in the interaction between CD14^+^ CD16^+^ monocyte cells, CD16^+^ monocyte cells, DCs, and HLA_DR^+^ Tregs from the severe COVID-19 patients, and the ligand-receptor pair of PF4_CXCR3 was not significantly enhanced in severe COVID-19 patients (**Figures 4F–H**). Therefore, the ligand-receptor pair of PF4_CXCR3 from the interaction between CD14^+^ monocytes and HLA_DR^+^ Tregs may contribute to the activation of HLA_DR^+^ Tregs in COVID-19 patients.

Furthermore, we found that a proportion of CD14^+^ monocyte cells was positively correlated with the inhibition of HLA_DR^+^ Tregs (R = 0.44, *P-value* = 0.0018), as the proportion of CD14^+^ monocyte cells increased, the suppression of HLA_DR^+^ Tregs increased (**Figure 4I**), while other myeloid cells were negatively correlated with the suppressive function of HLA_DR^+^ Tregs (**Supplementary Figures S5C–E**). In addition, the expression level of *CXCR3* of Tregs was significantly higher in severe COVID-19 patients than in the healthy controls in the bulk RNA-seq of Tregs (**Figure 4J**). Here, the proportion of CD14^+^ monocyte cells and the expression level of *CXCR3* of Tregs in severe COVID-19 patients supported that the ligand-receptor pair of PF4_CXCR3 promotes the activation of HLA_DR^+^ Tregs in severe COVID-19 patients.

Collectively, the ligand-receptor pair of PF4_CXCR3, in the interaction between CD14^+^ monocyte and HLA_DR^+^ Tregs, facilitates the suppression of HLA_DR^+^ Tregs in severe COVID-19 patients.

### Weakened TCR clonotype expansion of Tregs in COVID-19 patients

**Figure 5:**
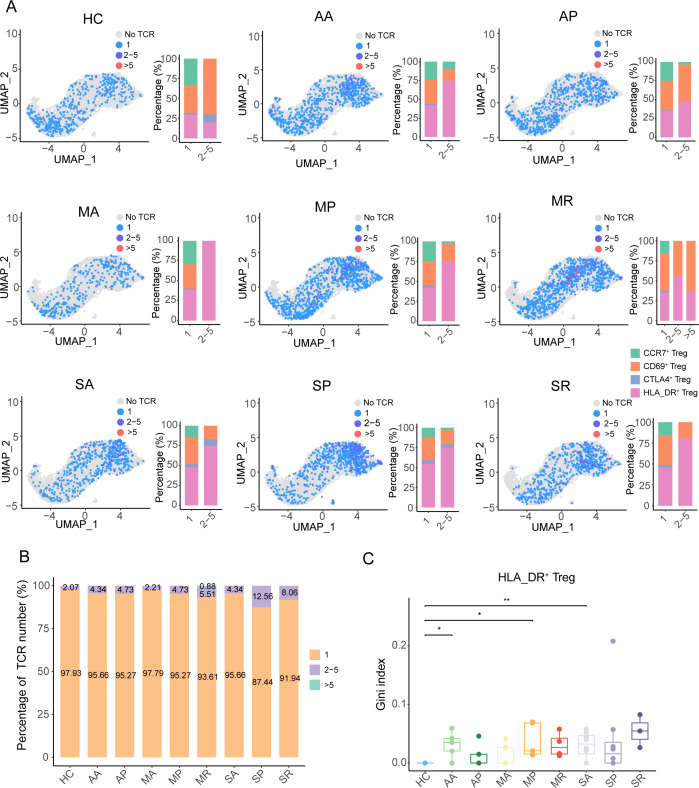
The TCR clonotype expansion feature of Tregs in COVID-19 patients. **(A)** The UMAP plots and histograms show the different sizes of TCR clonotypes corresponding to cell types in the eight COVID-19 patient groups and healthy controls. **(B)** The quantitative proportion of different types of TCR clonotypes in the eight COVID-19 patient groups and healthy controls. **(C)** Gini index distribution among the eight COVID-19 patient groups and healthy controls. *P-values* for pairwise comparisons were calculated by unpaired two-tailed Student’s t-test, *P < 0.05, **P < 0.01.

When examining the TCR diversity of Tregs, we found that only in the MR group was the size of some clonotypes greater than 5, while the largest clonotypes were less than 5 in other groups (**Figure 5A**). TCR clonotypes with sizes ranging from 2 to 5 were mainly concentrated in HLA_DR^+^ Tregs from the eight groups of COVID-19 patients, while in the healthy controls, these were enriched in the *CD69* ^+^ Tregs (**Figure 5A**). The percentage of TCR clonotype-expanded cells in all eight groups was less than 15% (**Figure 5B**). In addition, only the Gini index of TCR clonotypes from the AA, MP, and SA groups was significantly higher than that of the healthy controls (**Figure 5C**). Meanwhile, the Gini index of TCR clonotypes from severe COVID-19 patients was not significantly higher than the other groups (**Supplementary Figures S6A–D**). Thus, it can be observed that the TCR clonotypes in the eight groups of COVID-19 patients were rich in diversity and had low clonality.

**Figure 6:**
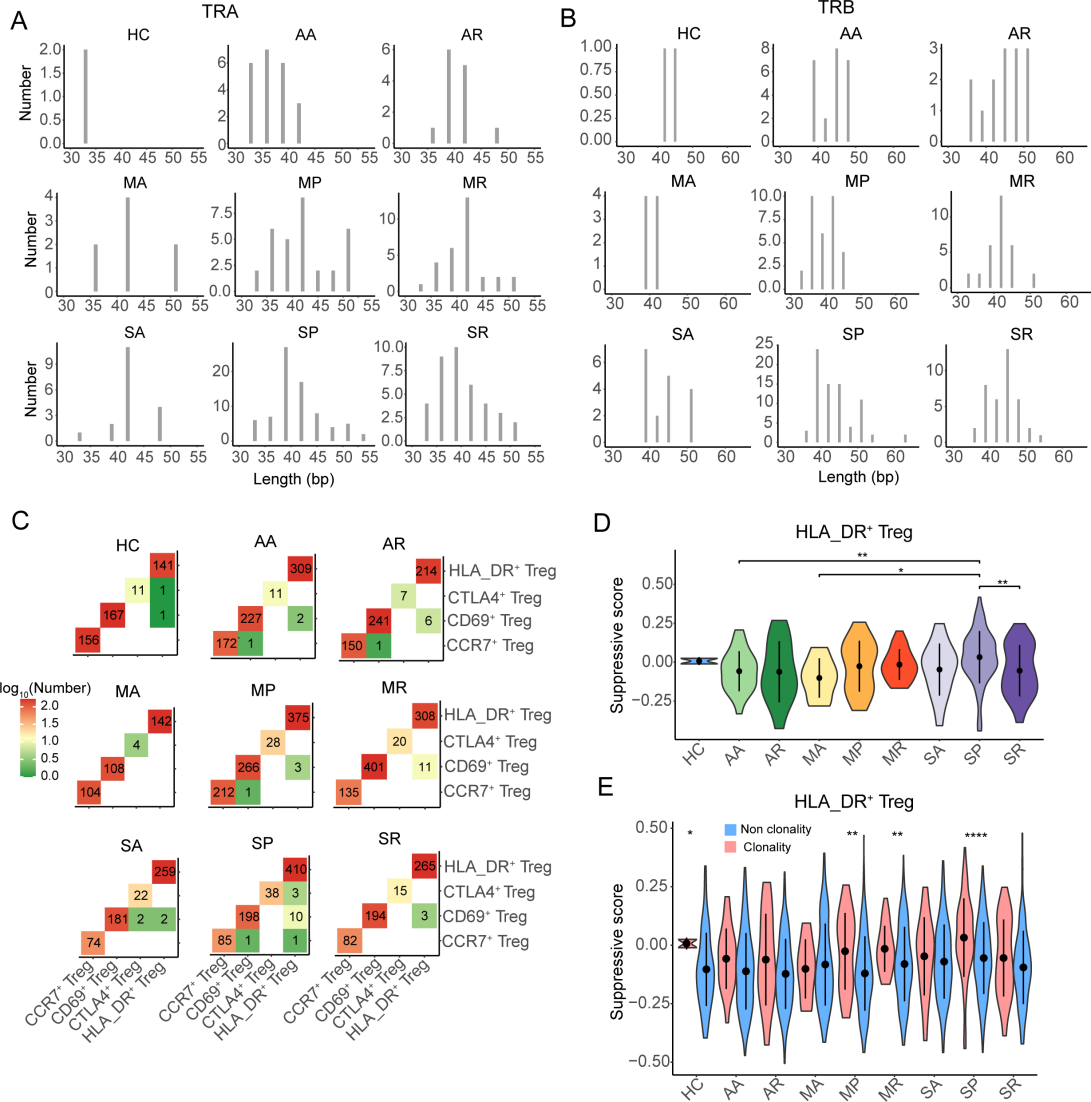
The TCR sharing and clonability of Tregs in COVID-19 patients. **(A, B)** The CDR3 nucleotide length distribution of TRA and TRB, respectively. **(C)** The TCR sharing among *CCR7* ^+^ Tregs, *CD69* ^+^ Tregs, *CTLA4* ^+^ Tregs, and *HLA-DR* ^+^ Tregs from eight COVID-19 patient groups and healthy controls. **(D)** The violin plot shows the distribution of suppressive scores among eight COVID-19 patient groups and healthy controls. **(E)** The distribution of suppressive score between non-clonality TCR clonotype and clonality TCR clonotype from eight COVID-19 patient groups and healthy controls. *P-values* for pairwise comparisons were calculated by unpaired two-tailed Student’s t-test, *P < 0.05, **P < 0.01, ****P < 0.0001.

Furthermore, we found that the eight groups of COVID-19 patients had different length distributions of CDR3 from TRA and TRB (**Figures 6A, B**), which might suggest that the TCR clonotypes from different groups recognized different antigens. There was no public TCR clonotype among the different populations of Tregs and across healthy controls and asymptomatic, mild, and severe patients. We also observed that there was no public TCR clonotype among the different populations of Tregs and across patients with different severities of COVID-19, and there was a higher frequency of TCR sharing between HLA_DR^+^ Tregs and *CD69* ^+^ Tregs (**Supplementary Figures S6E, F**), especially in the MR and SP groups (**Figure 6C**).

Moreover, we noted that the suppression of expanded TCRs of HLA_DR^+^ Tregs in the SP group was significantly higher than that in the AA, MA, and SR groups, while that in the other severe COVID-19 patients was not different from that in the other groups (**Figure 6D**). By comparing the suppression of clonotype-expanded TCRs with that of non-clonotype-expanded TCRs, we found that the suppression of clonotype-expanded TCRs was significantly higher than that of non-clonotype expansion only in the MP, MR, SP, and HC groups (**Figure 6E**).

Taken together, the clonability of the TCR clonotypes in the eight groups of COVID-19 patients was low, and the length of CDR3 had a different distribution. The suppression of expanded TCRs of HLA_DR^+^ Tregs in severe COVID-19 patients was not stronger than in the other groups.

## Discussion

Since the COVID-19 pandemic, numerous studies of peripheral blood from individuals infected with SARS-CoV-2 have revealed the proportion change and immune characteristics of Tregs from COVID-19 patients compared to healthy donors. However, the dynamic changes and heterogeneity of Tregs in COVID-19 patients are still lacking characterization. To fill this gap, we utilized single-cell transcriptomic technology to unveil the dynamic landscape of Tregs in COVID-19 patients with different disease severity and stage. We noted that the proportion of Tregs in healthy individuals was significantly lower compared to that in the AA, MP, MR, SA, and SP groups, but there was no significant difference when compared to the MA and SR groups. The percentage of Tregs in CD4^+^ T cells from the SA and SR groups showed a declining trend compared to the MP and MR groups but remained higher than in the MA group, while the proportion of Tregs in the SP group was quite similar to that in the MP and MR groups. This is consistent with previous observations of higher or lower proportions of Tregs in severe COVID-19 patients (27, 28, 40, 41). The proportion of Tregs at different disease stages in severe and mild cases showed varying trends compared to healthy individuals. Additionally, the ratio of Tregs at different disease stages in the mild cases had a different trend compared to that in the severe cases. Our results indicate that the proportion of Tregs varies within the same disease severity at different stages. Therefore, different studies might sample individuals at different stages of the same disease severity, leading to controversies regarding the variation of Tregs in COVID-19 patients. Further, we observed that the proportion of *CCR7* ^+^ Tregs in COVID-19 patients was lower than that in the healthy controls and decreased as the severity of the disease worsened. In contrast, the proportion of HLA_DR^+^ Tregs in COVID-19 patients was higher than that in healthy controls and increased as the severity of the disease worsened. Therefore, our study provided a longitudinal investigation of the characteristics of Tregs from COVID-19 patients.

In this study, we found that Tregs only have one developmental pathway, starting with *CCR7* ^+^ Tregs and ending with HLA_DR^+^ Tregs. Y Luo et al. categorized Tregs into two differentiation pathways with distinct phenotypic and functional programs in patients who underwent stem cell transplantation, ending with the FOXP3^hi^ and MKI67^hi^ subsets, respectively (42). Different diseases may induce different developmental pathways (42, 43). In addition, we found that *CARHSP1* was positively correlated with the proportion of HLA_DR^+^ Tregs, suggesting that *CARHSP1* positively regulated the development of HLA_DR^+^ Tregs. *CARHSP1* contains an S1-like cold-shock domain (CSD), making it possible for it to bind to polypyrimidine regions and regulate the stability of the target mRNA (44, 45). Miguel Lacal et al. reported that *CARHSP1* plays a role in the regulation of transcription after GPI treatment of endothelial cells (46). However, how *CARHSP1* regulates HLA_DR^+^ Tregs needs further study.

In this study, we found that HLA_DR^+^ Tregs exhibit stronger suppressive activity compared to other subsets of Tregs, with HLA_DR^+^ Tregs from severe cases showing the strongest suppressive function. Moreover, HLA_DR^+^ Tregs at the convalescent stage have greater suppressive function than other stages in mild COVID-19 patients, while HLA_DR^+^ Tregs at the progressive stage have greater suppressive function than other stages in severe COVID-19 patients. The inconsistent performance at different disease severities may have led to the controversy regarding Tregs (29).

Several possibilities have been proposed to induce the suppressive function of Tregs, including IL-16, IL-18, hypoxia, and high levels of lactic acid (30, 32, 33, 47, 48). However, we observed an enhanced interaction between *PF4* from CD14^+^ monocytes and *CXCR3* from HLA_DR^+^ Tregs in COVID-19 patients, suggesting that PF4-CXCR3 plays an important role in maintaining homeostasis and suppressive function of Tregs. *CXCR3* knockdown by siRNAs reduced *PF4*-enhanced Th1 and Treg responses and Tcm cell proliferation (49). Anti-*CXCL4* (*PF4*) antibody was found to inhibit the percentage of Tregs, while the recombinant *CXCL4* protein increased the percentage of Tregs in the CD4^+^ T in chronic osteomyelitis (50). *CXCR3* also serves as a receptor for *CXCL9*, *CXCL10*, and *CXCL11 (* 51, 52), but the enhanced interaction between *CXCR3* and these chemokines was undetectable in COVID-19 patients, as their protein and mRNA levels were very low or undetectable. Furthermore, we found that the proportion of CD14^+^ monocytes is positively correlated with the suppression of HLA_DR^+^ Tregs, and the proportion of CD14^+^ monocytes in severe COVID-19 patients is higher than in other COVID-19 patients. In addition, we found that the expression level of *CXCR3* in severe COVID-19 patients is higher than that in other COVID-19 patients. In summary, our study suggests that PF4-CXCR3 plays an important role in the suppressive function of Tregs in severe COVID-19 patients. Further, we explored the clonability and expansion of TCR in Tregs and found that HLA_DR^+^ Tregs has weak clonal expansion in COVID-19 patients, which suggests that the activation of HLA_DR^+^ Tregs in COVID-19 patients might be a SARS-CoV-2 non-specific induction.

Expanded TCR clonotypes in severe COVID-19 cases did not significantly increase compared to the asymptomatic and mild COVID-19 groups, and all COVID-19 patients had weak TCR clonotype expansion. The TCR clonotype of Tregs shows significant clonal expansion, suggesting that the activation of Tregs may be induced by specific antigens (53). Conversely, when the TCR clonal expansion of Tregs is relatively weak, it indicates that the activation of Tregs may be caused by bystander effects. Here, Tregs isolated through single-cell transcriptomics were non-antigen-specific Tregs and the TCR clonotype of Tregs showed weakened expansion, which suggested that the activation of Tregs may be driven by bystander effects.

There are some limitations to our study, the first being that we only focused on Tregs from PBMC samples of COVID-19 patients and not on tissue-specific responses. Tregs from COVID-19 patients were detected from all kinds of immune cells by bioinformatic methods and were not sorted from immune cells by flow cytometry. As a result, the cell yield from individuals was relatively low, making it challenging to capture rare Treg subsets, such as KI67^+^ Tregs, which might affect a comprehensive study of the immunological characteristics and function of Tregs. In addition, we cannot distinguish SARS-CoV-2-specific Tregs that truly reflect the immune response and function after infection with SARS-CoV-2.

In summary, we described a dynamic landscape of transcriptomics and functionality of Tregs in COVID-19 patients under different disease conditions, providing potential insights into factors that induce Treg activation in critically ill patients and offering a theoretical basis for the application of Tregs in the treatment of COVID-19 patients.

## Materials and methods

### Single-cell RNA-seq data pre-processing

The single-cell RNA data from 48 samples downloaded from the Beijing Institute of Genomics, Chinese Academy of Sciences (http://bigd.big.ac.cn/gsa-human) with accession number HRA000628 were used. The Cell Ranger toolkit (v 4.0.0) provided by 10× Genomics was used to align the sequencing data with the reference genome GRCh38 downloaded from 10× Genomics’ official website and generated the gene-cell unique molecular identifier (UMI) matrix. The mapping ratio of each sample was greater than 85% (**Supplementary Table S4**). For each cell, we quantified the number of genes and UMIs and kept high-quality cells with 200 to 5,500 genes detected and a mitochondrial gene count of no more than 12%.

### Unsupervised dimension reduction and cluster analysis

The filtered unique molecular identifiers of the genes were normalized using the “NormalizeData” program in the R Seurat package (v4.0.3) (54) with default parameters. Next, the “IntegrateData” function was applied to correct the batch effect between healthy donors and COVID-19 patients. Then, the “RunPCA” program was performed based on the top 2,000 highly variable genes generated by the “Find VariableFeatures” function, and the UMAP of single cells was generated using the “RunUMAP” program. Finally, we used “FindNeighbors” and “FindClusters” to cluster cells into sub-clusters at a resolution of 0.8 and visualized them by UMAP with default settings.

According to the specific markers (*CD3E*, *CD3D*, and *CD3B*), all T cells were singled out from the clustered cells. T cells from healthy donors and the eight groups of COVID-19 patients were re-clustered following the steps described above, including integration, dimension reduction, and clustering analysis. Then, CD4^+^ T cells were chosen with high levels of expression of *CD4* genes and low expression of *CD8A* and *CD8B* genes and were re-clustered into different sub-clusters. Approximately 7,873 Tregs were selected with high expression of *FOXP3* and *IL2AR* genes. The CD4^+^ Tregs were further re-clustered into sub-clusters with a resolution of 0.6, resulting in eight clusters. Finally, the CD4^+^ Tregs were ultimately categorized into four subpopulations based on the highly expressed characteristic genes in each cluster.

### Identification of marker genes and annotation of cell clusters

Marker genes for each cluster were identified with the MAST algorithm in the FindAllMarkers function of Seurat. The following criteria were used to filter the markers: |log_2_FC| >=0.585, *p.adjust* <= 0.05, and pct.1>0.25. The cell clusters were annotated using previously reported cell type-specific marker genes.

### Calculating gene expression signature scores

The cell gene expression signature score was calculated using the “AddModuleScore” function in Seurat. This function operates as follows. It computes the average expression values of genes within each specified gene set for each cell and selects a background gene set that is comparable in size to the target gene set. The gene module score is then obtained by subtracting the average expression values of the background gene set from those of the target gene set. The background gene set is generated by a function that randomly selects genes from multiple partitions of the expression matrix. The suppressive scores of the Tregs were calculated using the following genes: *NT5E*, *FGL2*, *CTLA4*, *ENTPD1*, *TGFB1*, *LGALS1*, *EBI3*, *IL12A*, *PRF1*, *GZMA*, *GZMB*, *IL2RA*, and IL10 (30). The suppressive score represents the suppressive strength of the Tregs. The higher the score, the stronger the suppression. The HLA_DR scores of the Tregs were then calculated using the genes as follows: *HLA_DRB5*, *HLA-DQA1*, *HLA_DRB1*, *HLA-DPA1*, *HLA-DPB1*, *HLA-DQB1*, and *HLA_DRA*.

### Construction of single-cell trajectories

Two methods were used to construct the single-cell trajectories, namely Monocle3 (55) and slingshot (56). Using Monocle3, the Tregs identified in the Seurat clustering analysis were fitted to a principal graph within each partition using the “learn_graph” program. Subsequently, based on their progression through the developmental program, the cells were ordered with *CCR7* ^+^ Tregs as the root cells. Pseudo-temporal was then employed to quantify the developmental process. Using the slingshot program, the expression matrix of the cell embeddings of all Tregs was utilized as input to generate inferred trajectories. Following the pseudo-temporal order established in Monocle3, Tregs were segmented into three equal parts, comprising early, middle, and later stages. The top 20 genes with high expression at the early and later stages were visualized in a heatmap plot. Additionally, differentially expressed transcription factors at the early and later stages across various groups were depicted in early bubble heatmap plots.

### Functional annotation analysis

The “FindMarkers” function was applied to detect the differentially expressed genes (DEGs) from a pair-wise comparison. The following criteria were used to define DEGs: |log_2_FC|≥0.26, *P-value ≤* 0.05, and pct.1>0.25. Gene Ontology (GO) and Kyoto Encyclopedia of Genes and Genomes (KEGG) pathway analyses of the DEGs were performed using the clusterProfiler R package (57), and only terms in the “GO Biological Processes” were considered in the GO enrichment analysis. In addition, GSEA was also included and performed with C5 (Gene Ontology) in MSigDB (58).

### Protein-protein interactions

The “FindMarkers” function was used to define the differentially expressed genes that were upregulated in HLA_DR^+^ Tregs compared to other cell clusters. The following criteria were used to define DEGs: log_2_FC≥0.26, *P-value ≤* 0.05, and pct.1>0.25 Subsequently, a protein-protein interaction network was constructed using STRING. Genes interacting with *CARHSP1* and their downstream genes were selected, and then a sub-protein interaction network was constructed using STRING.

### Cell–cell interaction analysis using CellPhoneDB

Curated receptors, ligands, and their interactions are stored in CellPhoneDB (Version 2.1.2) (59), which is a publicly available repository. CellPhoneDB allows searching for particular ligands/receptors or interrogating single-cell transcriptomic data. Aiming to reveal the cell-cell interactions between the HCs and the eight groups of COVID-19 patients, CellphoneDB in Python (version 3.6.0) was used with default parameters. Furthermore, significant interaction pairs (*P-value* <= 0.05) were reserved for the subsequent analyses. According to the annotation in CellPhoneDB, the genes in the cell-cell interactions were separated into ligands and receptors for further studies. The genes annotated as “True” receptors in the interacting pair were accepted as receptors interacting in the cell-cell interactions. The “False” annotation was taken as a ligand. Ligand-derived cell types were treated as regulatory cells (source cell types), while the receptor-derived cell types were regarded as regulated cells (target cell types). For downstream analysis, we counted and compared cell-cell interactions in 28 clusters from the HCs and the eight groups of COVID-19 patients and then selected the most variable cellular interactions.

### Single-cell TCR analysis

The nucleotide and amino acid sequences of TCR chains were assembled and annotated using the Cell Ranger vdj program (version 4.0.0), and the proportion of reads that were mapped to the V(D)J region exceeded 70% in most samples (**Supplementary Table S5**). Only cells with paired TRA and TRB were used in the follow-up analysis. An exact match of amino acid sequences of CDR3 and matching V and J genes of both TRA and TRB was defined as the same TCR clonotype. Subsequently, the barcodes of Tregs from the single-cell transcriptome were matched with the barcodes of the retained TCR clonotypes to obtain the TCR clonotype cell types. The analysis of clonotype expansion and the length of CDR3 was performed for the TCR clonotypes with cell type annotation. Finally, there were 5,973 TCRs with annotated cell types. The number of different TCR clonotypes of different cell types in each group is listed in **Supplementary Table S6**. Statistical analysis of the Gini index was used to measure the cross-compartment clonal diversity. The Gini index was calculated using the Gini function of the reldist R package (60). A two-tailed Student’s t-test was used to detect the significance of the differences.

### RNA-seq analysis

The gene count representing the gene expression level of Tregs from bulk RNA-seq was downloaded from the Gene Expression Omnibus (GEO) database with accession no. GSE179448, including 13 severe COVID-19 patients, 6 mild COVID-19 patients, 19 recovered COVID-19 patients, and 7 healthy controls. Detailed clinical information is presented in **Supplementary Table S7**. The R package DESeq2 (61) was used to compute the DEGs between the different groups from the normalized read count dataset. Genes with FoldChange≥1 or ≤-1 and *P-value* ≤ 0.05 were selected as DEGs.

### Study approval

The study protocol conformed to the ethical guidelines of the 1975 Declaration of Helsinki, as reflected by the *a priori* approval of the institution’s human research committee (Ethics Committee of Renji Hospital). Written informed consent was obtained from the parents of each patient included in the study. No donor organs were obtained from executed prisoners or other institutionalized persons.

## Data Availability

The original contributions presented in the study are included in the article/**Supplementary Material**. Further inquiries can be directed to the corresponding author.

## Supplementary material

Supplementary Figure 1 Selection of the Tregs. **(A)** The UMAP plot shows the subcluster of CD4^+^ T cells from COVID-19 patients and healthy controls. **(B)** The UMAP plot shows the subcluster of Tregs. **(C, D)** The UMAP plots show the expression distribution of key markers related to Tregs in the CD4^+^ T cells **(C)** and Tregs**(D)**.

Supplementary Figure 2 The pseudo-temporal trajectory of Tregs from COVID-19 patients at the stage of acute, progress, recovery period, and healthy controls. **(A, B)** Pseudo-temporal trajectory of Tregs from COVID-19 patients at the stage of acute progression, recovery period, and healthy controls by Slingshot **(A)** andMonocle3 **(B)**. **(C, D)** Pseudo-temporal developmental trajectory of Tregs from asymptomatic, mild, severe COVID-19 patients and healthy controls by slingshot **(A)** and monocle3 **(B)**. **(E)** The protein-protein interactions between *CARHSP1* and other genes.

Supplementary Figure 3 Immune characteristics of all Tregs, *CCR7* ^+^ Tregs, *CD69* ^+^ Tregs, and *CTLA4* ^+^ Tregs. **(A–D)** The violin plot shows the suppressive score of eight COVID-19 patient groups and healthy controls in all Tregs **(A)**, *CCR7* ^+^ Tregs **(B)**, *CD69* ^+^ Tregs **(C)**, and *CTLA4* ^+^ Tregs **(D)**. **(E, H)** The violin plot shows the suppressive score of eight COVID-19 patient groups and healthy controls in all Tregs **(E)**, *CCR7* ^+^ Tregs **(F)**, *CD69* ^+^ Tregs **(G)**, and *CTLA4* ^+^ Tregs **(H)**. **(I)** The point plots show the pairwise comparison of differentially expressed genes in Tregs between HC and COVID-19 patients at the recovery stage. *P-values* for pairwise comparisons were calculated by unpaired two-tailed Student’s t-test, *P < 0.05, **P < 0.01, ***P < 0.001, ****P < 0.0001.

Supplementary Figure 4 The expression distribution of selected genes is associated with cytokine, chemokine receptor, chemokine ligand, TNF family ligand, and TNF family receptor. **(A–D)** The bubble heatmap plot shows the expression level of selected genes associated with cytokine, chemokine receptor, chemokine ligand, TNF family ligand, TNF family receptor in all Tregs **(A)**, *CCR7* ^+^ Tregs **(B)**, *CD69* ^+^ Tregs **(C)** and *CTLA4* ^+^ Tregs **(D)**.

Supplementary Figure 5 Cellular populations of immune cells in PBMC samples from COVID-19 patients and healthy controls. **(A)** The UMAP plot displays the distribution of immune cells, including NK/NKT cells, T cells, B cells, myeloid cells **(B)** The heatmap shows the expression level of cell-specific markers for cell clusters. **(C–E)** The correlation between the suppressive score of HLA-DR^+^ Tregs and the cell percentage of DC **(C)**, CD14^+^ CD16^+^ monocytes **(D)**, and CD16^+^ monocytes **(E)**.

Supplementary Figure 6 The distribution of the Gini index in Tregs. **(A–D)** Distribution of the Gini index in all Tregs **(A)**, *CCR7* ^+^ Tregs **(B)**, *CD69* ^+^ Tregs **(C)**, and *CTLA4* ^+^ Tregs **(D)**. **(E, F)** The sharing of TCRs among different Treg cell subpopulations **(E)** and across various disease stages **(F)**. *P-values* for pairwise comparisons were calculated by unpaired two-tailed Student’s t-test, *P < 0.05, **P < 0.01, ***P < 0.001, ****P < 0.0001.

## References

[1] Hori S Nomura T Sakaguchi S. Control of regulatory T cell development by the transcription factor foxp3. Science. (2003) 299:1057–61. doi: 10.1126/science.1079490 12522256

[2] Dominguez-Villar M Hafler DA. Regulatory T cells in autoimmune disease. Nat Immunol. (2018) 19:665–73. doi: 10.1038/s41590-018-0120-4 PMC7882196 29925983

[3] Togashi Y Shitara K Nishikawa H. Regulatory T cells in cancer immunosuppression - implications for anticancer therapy. Nat Rev Clin Oncol. (2019) 16:356–71. doi: 10.1038/s41571-019-0175-7 30705439

[4] Vignali DA Collison LW Workman CJ. How regulatory T cells work. Nat Rev Immunol. (2008) 8:523–32. doi: 10.1038/nri2343 PMC2665249 18566595

[5] Asseman C Mauze S Leach MW Coffman RL Powrie F. An essential role for interleukin 10 in the function of regulatory T cells that inhibit intestinal inflammation. J Exp Med. (1999) 190:995–1004. doi: 10.1084/jem.190.7.995 10510089 PMC2195650

[6] Powrie F Carlino J Leach MW Mauze S Coffman RL. A critical role for transforming growth factor-beta but not interleukin 4 in the suppression of T helper type 1-mediated colitis by cd45rb(Low) cd4+ T cells. J Exp Med. (1996) 183:2669–74. doi: 10.1084/jem.183.6.2669 PMC2192626 8676088

[7] Curotto de Lafaille MA Lafaille JJ. Natural and adaptive foxp3+ Regulatory T cells: more of the same or a division of labor? Immunity. (2009) 30:626–35. doi: 10.1016/j.immuni.2009.05.002 19464985

[8] Sakaguchi S Yamaguchi T Nomura T Ono M. Regulatory T cells and immune tolerance. Cell. (2008) 133:775–87. doi: 10.1016/j.cell.2008.05.009 18510923

[9] Knoechel B Lohr J Kahn E Bluestone JA Abbas AK. Sequential development of interleukin 2-dependent effector and regulatory T cells in response to endogenous systemic antigen. J Exp Med. (2005) 202:1375–86. doi: 10.1084/jem.20050855 PMC2212975 16287710

[10] Schulte-Schrepping J Reusch N Paclik D Baßler K Schlickeiser S Zhang B. Severe covid-19 is marked by a dysregulated myeloid cell compartment. Cell. (2020) 182:1419–40.e23. doi: 10.1016/j.cell.2020.08.001 32810438 PMC7405822

[11] Sacks D Baxter B Campbell BCV Carpenter JS Cognard C Dippel D. Multisociety consensus quality improvement revised consensus statement for endovascular therapy of acute ischemic stroke. Int J Stroke. (2018) 13:612–32. doi: 10.1177/1747493018778713 29786478

[12] Kuri-Cervantes L Pampena MB Meng W Rosenfeld AM Ittner CAG Weisman AR. Comprehensive mapping of immune perturbations associated with severe covid-19. Sci Immunol. (2020) 5:eabd7114. doi: 10.1126/sciimmunol.abd7114 32669287 PMC7402634

[13] Leisman DE Ronner L Pinotti R Taylor MD Sinha P Calfee CS. Cytokine elevation in severe and critical covid-19: A rapid systematic review, meta-analysis, and comparison with other inflammatory syndromes. Lancet Respir Med. (2020) 8:1233–44. doi: 10.1016/s2213-2600(20)30404-5 PMC7567529 33075298

[14] Remy KE Mazer M Striker DA Ellebedy AH Walton AH Unsinger J. Severe immunosuppression and not a cytokine storm characterizes covid-19 infections. JCI Insight. (2020) 5:e140329. doi: 10.1172/jci.insight.140329 32687484 PMC7526441

[15] Cao X. Covid-19: immunopathology and its implications for therapy. Nat Rev Immunol. (2020) 20:269–70. doi: 10.1038/s41577-020-0308-3 PMC7143200 32273594

[16] Wang F Hou H Luo Y Tang G Wu S Huang M. The laboratory tests and host immunity of covid-19 patients with different severity of illness. JCI Insight. (2020) 5:e137799. doi: 10.1172/jci.insight.137799 32324595 PMC7259533

[17] Vigón L Fuertes D García-Pérez J Torres M Rodríguez-Mora S Mateos E. Impaired cytotoxic response in pbmcs from patients with covid-19 admitted to the icu: biomarkers to predict disease severity. Front Immunol. (2021) 12:665329. doi: 10.3389/fimmu.2021.665329 34122423 PMC8187764

[18] Jiang M Guo Y Luo Q Huang Z Zhao R Liu S. T-cell subset counts in peripheral blood can be used as discriminatory biomarkers for diagnosis and severity prediction of coronavirus disease 2019. J Infect Dis. (2020) 222:198–202. doi: 10.1093/infdis/jiaa252 32379887 PMC7239156

[19] Chen X Huang J Huang Y Chen J Huang Y Jiang X. Characteristics of immune cells and cytokines in patients with coronavirus disease 2019 in guangzhou, China. Hum Immunol. (2020) 81:702–8. doi: 10.1016/j.humimm.2020.08.006 PMC7495147 32950268

[20] De Biasi S Meschiari M Gibellini L Bellinazzi C Borella R Fidanza L. Marked T cell activation, senescence, exhaustion and skewing towards th17 in patients with covid-19 pneumonia. Nat Commun. (2020) 11:3434. doi: 10.1038/s41467-020-17292-4 32632085 PMC7338513

[21] Del Bello A Kamar N Vergez F Faguer S Marion O Beq A. Adaptive lymphocyte profile analysis discriminates mild and severe forms of covid-19 after solid organ transplantation. Kidney Int. (2021) 100:915–27. doi: 10.1016/j.kint.2021.05.032 PMC8193964 34126110

[22] Laing AG Lorenc A Del Molino Del Barrio I Das A Fish M Monin L. A dynamic covid-19 immune signature includes associations with poor prognosis. Nat Med. (2020) 26:1623–35. doi: 10.1038/s41591-020-1038-6 32807934

[23] Tan M Liu Y Zhou R Deng X Li F Liang K. Immunopathological characteristics of coronavirus disease 2019 cases in guangzhou, China. Immunology. (2020) 160:261–8. doi: 10.1111/imm.13223 PMC7283723 32460357

[24] Mohebbi SR Baghaei K Rostami-Nejad M Nazemalhosseini Mojarad E Mirjalali H Yadegar A. Significant changes of cd4, foxp3, cd25, and il6 expression level in Iranian covid-19 patients. Gastroenterol Hepatol Bed Bench. (2020) 13:388–92. PMC7682958 33244382

[25] Qin C Zhou L Hu Z Zhang S Yang S Tao Y. Dysregulation of immune response in patients with coronavirus 2019 (Covid-19) in wuhan, China. Clin Infect diseases: an Off Publ Infect Dis Soc America. (2020) 71:762–8. doi: 10.1093/cid/ciaa248 PMC7108125 32161940

[26] Kang CK Han GC Kim M Kim G Shin HM Song KH. Aberrant hyperactivation of cytotoxic T-cell as a potential determinant of covid-19 severity. Int J Infect diseases: IJID: Off Publ Int Soc Infect Dis. (2020) 97:313–21. doi: 10.1016/j.ijid.2020.05.106 PMC7261468 32492530

[27] Meckiff BJ Ramírez-Suástegui C Fajardo V Chee SJ Kusnadi A Simon H. Imbalance of regulatory and cytotoxic sars-cov-2-reactive cd4(+) T cells in covid-19. Cell. (2020) 183:1340–53.e16. doi: 10.1016/j.cell.2020.10.001 33096020 PMC7534589

[28] Mahmoud Salehi Khesht A Karpisheh V Qubais Saeed B Olegovna Zekiy A Yapanto LM Nabi Afjadi M. Different T cell related immunological profiles in covid-19 patients compared to healthy controls. Int Immunopharmacol. (2021) 97:107828. doi: 10.1016/j.intimp.2021.107828 34091116 PMC8162824

[29] Xu Z Jiang X Dai X Li B. The dynamic role of foxp3(+) tregs and their potential therapeutic applications during sars-cov-2 infection. Front Immunol. (2022) 13:916411. doi: 10.3389/fimmu.2022.916411 35874688 PMC9305488

[30] Galván-Peña S Leon J Chowdhary K Michelson DA Vijaykumar B Yang L. Profound treg perturbations correlate with covid-19 severity. Proc Natl Acad Sci U.S.A. (2021) 118:e2111315118. doi: 10.1073/pnas.2111315118 34433692 PMC8449354

[31] Vick SC Frutoso M Mair F Konecny AJ Greene E Wolf CR. A regulatory T cell signature distinguishes the immune landscape of covid-19 patients from those with other respiratory infections. Sci Adv. (2021) 7:eabj0274. doi: 10.1126/sciadv.abj0274 34757794 PMC8580318

[32] Dhont S Derom E Van Braeckel E Depuydt P Lambrecht BN. The pathophysiology of ‘Happy’ Hypoxemia in covid-19. Respir Res. (2020) 21:198. doi: 10.1186/s12931-020-01462-5 32723327 PMC7385717

[33] McElvaney OJ McEvoy NL McElvaney OF Carroll TP Murphy MP Dunlea DM. Characterization of the inflammatory response to severe covid-19 illness. Am J Respir Crit Care Med. (2020) 202:812–21. doi: 10.1164/rccm.202005-1583OC PMC7491404 32584597

[34] Xu G Qi F Wang H Liu Y Wang X Zou R. The transient ifn response and the delay of adaptive immunity feature the severity of covid-19. Front Immunol. (2021) 12:816745. doi: 10.3389/fimmu.2021.816745 35095917 PMC8795972

[35] Hu H Miao YR Jia LH Yu QY Zhang Q Guo AY. Animaltfdb 3.0: A comprehensive resource for annotation and prediction of animal transcription factors. Nucleic Acids Res. (2019) 47:D33–d8. doi: 10.1093/nar/gky822 PMC6323978 30204897

[36] Bonnin E Rodrigo Riestra M Marziali F Mena Osuna R Denizeau J Maurin M. Cd74 supports accumulation and function of regulatory T cells in tumors. Nat Commun. (2024) 15:3749. doi: 10.1038/s41467-024-47981-3 38702311 PMC11068745

[37] Yang S Xie C Chen Y Wang J Chen X Lu Z. Differential roles of tnfα-tnfr1 and tnfα-tnfr2 in the differentiation and function of cd4(+)Foxp3(+) induced treg cells *in vitro* and *in vivo* periphery in autoimmune diseases. Cell Death Dis. (2019) 10:27. doi: 10.1038/s41419-018-1266-6 30631042 PMC6328545

[38] Tracey KJ Vlassara H Cerami A. Cachectin/tumour necrosis factor. Lancet (London England). (1989) 1:1122–6. doi: 10.1016/s0140-6736(89)92394-5 2566060

[39] Bradley JR. Tnf-mediated inflammatory disease. J Pathol. (2008) 214:149–60. doi: 10.1002/path.2287 18161752

[40] Caldrer S Mazzi C Bernardi M Prato M Ronzoni N Rodari P. Regulatory T cells as predictors of clinical course in hospitalised covid-19 patients. Front Immunol. (2021) 12:789735. doi: 10.3389/fimmu.2021.789735 34925369 PMC8674838

[41] Sadeghi A Tahmasebi S Mahmood A Kuznetsova M Valizadeh H Taghizadieh A. Th17 and treg cells function in sars-cov2 patients compared with healthy controls. J Cell Physiol. (2021) 236:2829–39. doi: 10.1002/jcp.30047 32926425

[42] Luo Y Xu C Wang B Niu Q Su X Bai Y. Single-cell transcriptomic analysis reveals disparate effector differentiation pathways in human T(Reg) compartment. Nat Commun. (2021) 12:3913. doi: 10.1038/s41467-021-24213-6 34162888 PMC8222404

[43] Hui Z Zhang J Zheng Y Yang L Yu W An Y. Single-cell sequencing reveals the transcriptome and tcr characteristics of ptregs and *in vitro* expanded itregs. Front Immunol. (2021) 12:619932. doi: 10.3389/fimmu.2021.619932 33868236 PMC8044526

[44] Schäfer C Steffen H Krzykowski KJ Göke B Groblewski GE. Crhsp-24 phosphorylation is regulated by multiple signaling pathways in pancreatic acinar cells. Am J Physiol Gastrointest Liver Physiol. (2003) 285:G726–34. doi: 10.1152/ajpgi.00111.2003 12801884

[45] Hou H Wang F Zhang W Wang D Li X Bartlam M. Structure-functional analyses of crhsp-24 plasticity and dynamics in oxidative stress response. J Biol Chem. (2011) 286:9623–35. doi: 10.1074/jbc.M110.177436 PMC3058955 21177848

[46] Lacal PM Tentori L Muzi A Ruffini F Dorio AS Xu W. Pharmacological inhibition of poly(Adp-ribose) polymerase activity down-regulates the expression of syndecan-4 and id-1 in endothelial cells. Int J Oncol. (2009) 34:861–72. doi: 10.3892/ijo_00000213 19212692

[47] Facciabene A Peng X Hagemann IS Balint K Barchetti A Wang LP. Tumour hypoxia promotes tolerance and angiogenesis via ccl28 and T(Reg) cells. Nature. (2011) 475:226–30. doi: 10.1038/nature10169 21753853

[48] Watson MJ Vignali PDA Mullett SJ Overacre-Delgoffe AE Peralta RM Grebinoski S. Metabolic support of tumour-infiltrating regulatory T cells by lactic acid. Nature. (2021) 591:645–51. doi: 10.1038/s41586-020-03045-2 PMC7990682 33589820

[49] Tan S Zhang J Sun Y Gisterå A Sheng Z Malmström RE. Platelets enhance cd4+ Central memory T cell responses via platelet factor 4-dependent mitochondrial biogenesis and cell proliferation. Platelets. (2022) 33:360–70. doi: 10.1080/09537104.2021.1936479 34137652

[50] Huang K Ge S. The anti-cxcl4 antibody depletes cd4(+)Cd25(+)Foxp3(+) regulatory T cells in cd4+ T cells from chronic osteomyelitis patients by the stat5 pathway. Ann Palliat Med. (2020) 9:2723–30. doi: 10.21037/apm-20-166 32954739

[51] Groom JR Luster AD. Cxcr3 ligands: redundant, collaborative and antagonistic functions. Immunol Cell Biol. (2011) 89:207–15. doi: 10.1038/icb.2010.158 PMC3863330 21221121

[52] Satarkar D Patra C. Evolution, expression and functional analysis of cxcr3 in neuronal and cardiovascular diseases: A narrative review. Front Cell Dev Biol. (2022) 10:882017. doi: 10.3389/fcell.2022.882017 35794867 PMC9252580

[53] Mair F Erickson JR Frutoso M Konecny AJ Greene E Voillet V. Extricating human tumour immune alterations from tissue inflammation. Nature. (2022) 605:728–35. doi: 10.1038/s41586-022-04718-w PMC9132772 35545675

[54] Hao Y Hao S Andersen-Nissen E Mauck WM 3rd Zheng S Butler A. Integrated analysis of multimodal single-cell data. Cell. (2021) 184:3573–87.e29. doi: 10.1016/j.cell.2021.04.048 34062119 PMC8238499

[55] Cao J Spielmann M Qiu X Huang X Ibrahim DM Hill AJ. The single-cell transcriptional landscape of mammalian organogenesis. Nature. (2019) 566:496–502. doi: 10.1038/s41586-019-0969-x 30787437 PMC6434952

[56] Street K Risso D Fletcher RB Das D Ngai J Yosef N. Slingshot: cell lineage and pseudotime inference for single-cell transcriptomics. BMC Genomics. (2018) 19:477. doi: 10.1186/s12864-018-4772-0 29914354 PMC6007078

[57] Yu G Wang LG Han Y He QY. Clusterprofiler: an R package for comparing biological themes among gene clusters. Omics: J Integr Biol. (2012) 16:284–7. doi: 10.1089/omi.2011.0118 PMC3339379 22455463

[58] Liberzon A Birger C Thorvaldsdóttir H Ghandi M Mesirov JP Tamayo P. The molecular signatures database (Msigdb) hallmark gene set collection. Cell Syst. (2015) 1:417–25. doi: 10.1016/j.cels.2015.12.004 PMC4707969 26771021

[59] Efremova M Vento-Tormo M Teichmann SA Vento-Tormo R. Cellphonedb: inferring cell-cell communication from combined expression of multi-subunit ligand-receptor complexes. Nat Protoc. (2020) 15:1484–506. doi: 10.1038/s41596-020-0292-x 32103204

[60] Brown MC. Using gini-style indices to evaluate the spatial patterns of health practitioners: theoretical considerations and an application based on alberta data. Soc Sci Med. (1994) 38:1243–56. doi: 10.1016/0277-9536(94)90189-9 8016689

[61] Love MI Huber W Anders S. Moderated estimation of fold change and dispersion for rna-seq data with deseq2. Genome Biol. (2014) 15:550. doi: 10.1186/s13059-014-0550-8 25516281 PMC4302049

