# Supplementary figures and images for: Heterogeneity and longitudinal transcriptomic characteristics of Tregs in COVID-19 patients
Source: Front Immunol. 2025 Mar 6;16:1548173. doi: 10.3389/fimmu.2025.1548173 (PMC11922936; doi:10.3389/fimmu.2025.1548173)

Fig S1

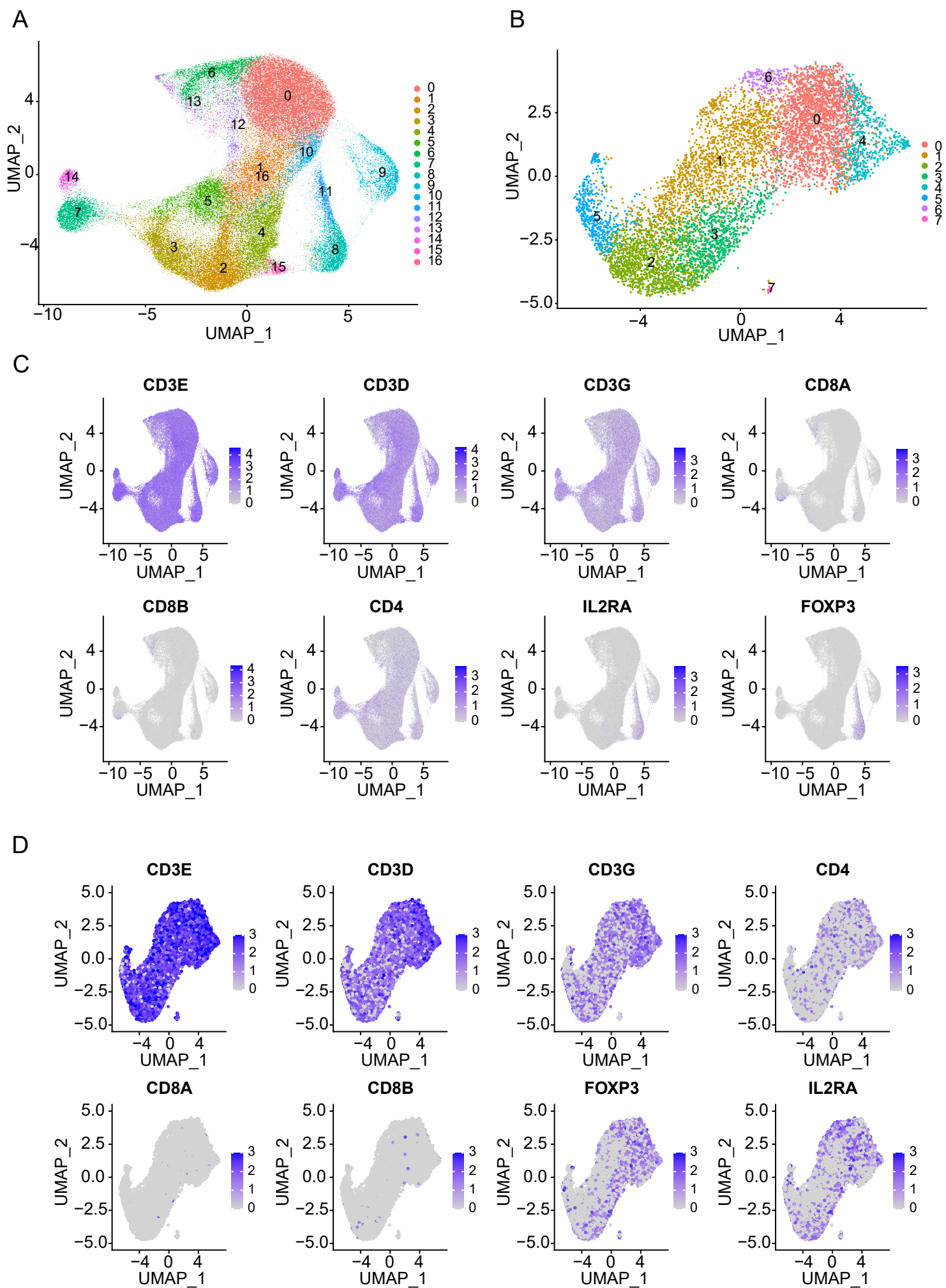

Supplement: Supplementary Figure 1 — Selection of the Tregs. (A) The UMAP plot shows the subcluster of CD4+ T cells from COVID-19 patients and healthy controls. (B) The UMAP plot shows the subcluster of Tregs. (C, D) The UMAP plots show the expression distribution of key markers related to Tregs in the CD4+ T cells (C) and Tregs(D). [file SupplementaryFile1.pdf]

Fig S2

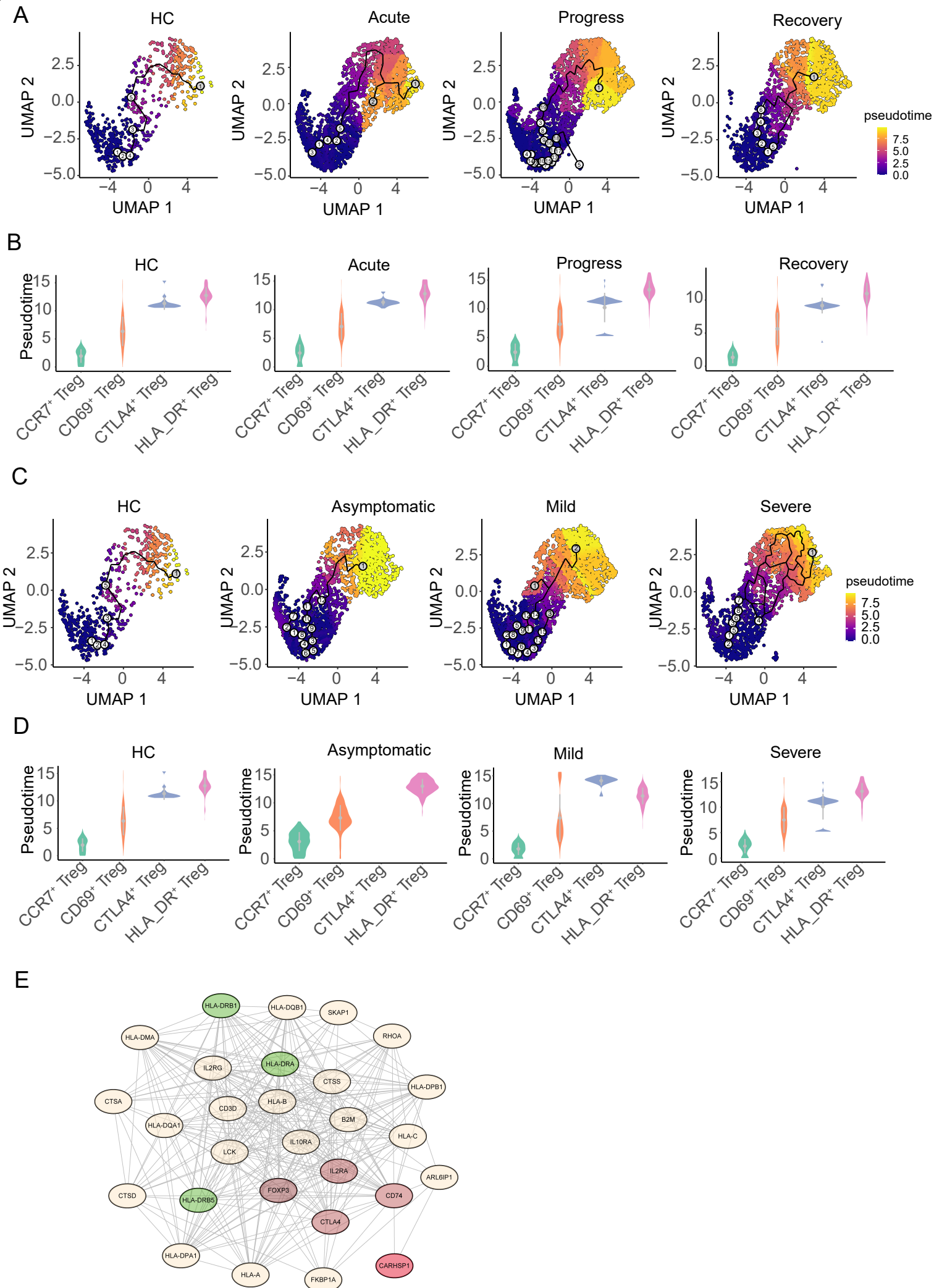

Supplement: Supplementary Figure 2 — The pseudo-temporal trajectory of Tregs from COVID-19 patients at the stage of acute, progress, recovery period, and healthy controls. (A, B) Pseudo-temporal trajectory of Tregs from COVID-19 patients at the stage of acute progression, recovery period, and healthy controls by Slingshot (A) andMonocle3 (B). (C, D) Pseudo-temporal developmental trajectory of Tregs from asymptomatic, mild, severe COVID-19 patients and healthy controls by slingshot (A) and monocle3 (B). (E) The protein-protein interactions between CARHSP1 and other genes. [file SupplementaryFile2.pdf]

Fig S3

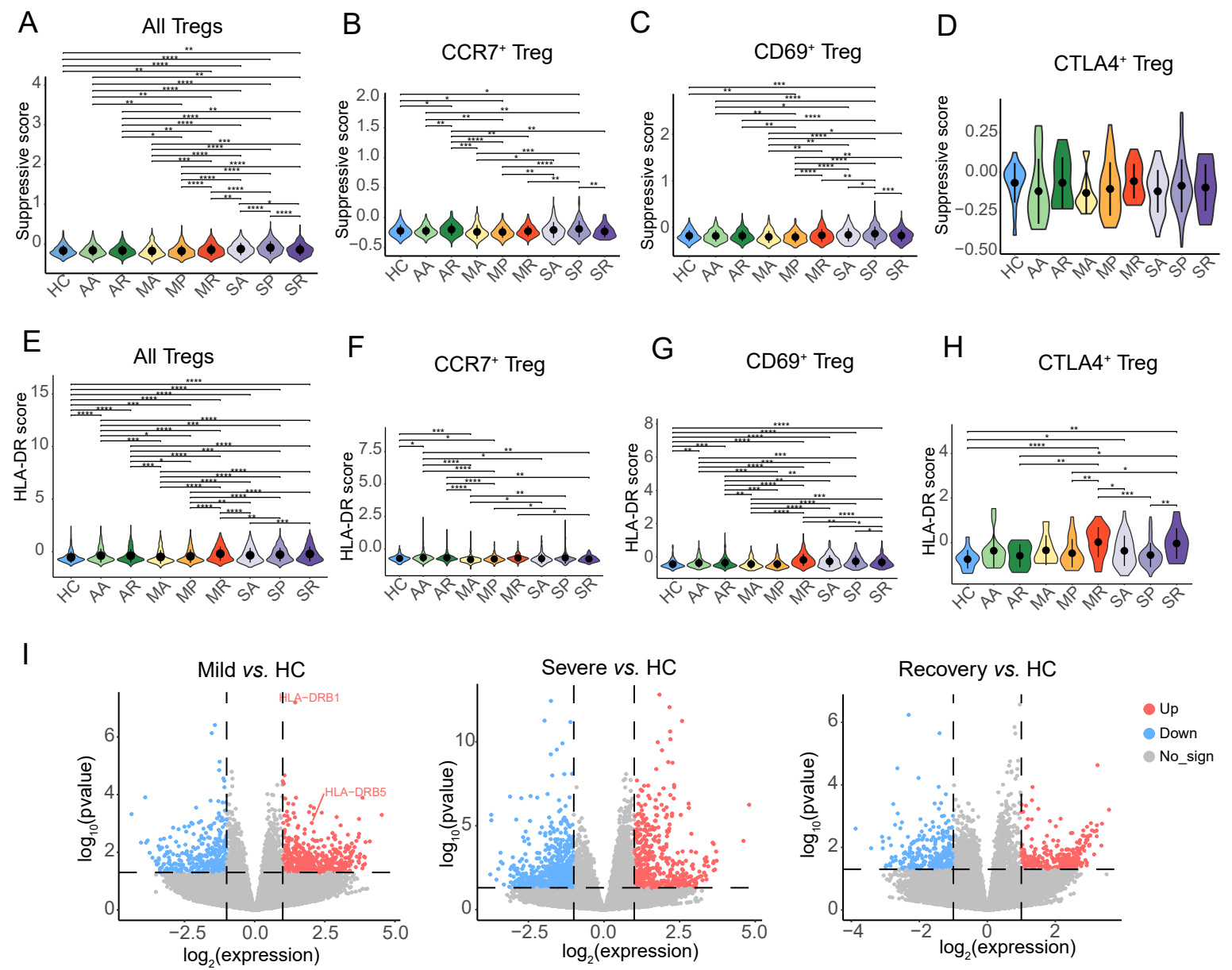

Supplement: Supplementary Figure 3 — Immune characteristics of all Tregs, CCR7 + Tregs, CD69 + Tregs, and CTLA4 + Tregs. (A–D) The violin plot shows the suppressive score of eight COVID-19 patient groups and healthy controls in all Tregs (A), CCR7 + Tregs (B), CD69 + Tregs (C), and CTLA4 + Tregs (D). (E, H) The violin plot shows the suppressive score of eight COVID-19 patient groups and healthy controls in all Tregs (E), CCR7 + Tregs (F), CD69 + Tregs (G), and CTLA4 + Tregs (H). (I) The point plots show the pairwise comparison of differentially expressed genes in Tregs between HC and COVID-19 patients at the recovery stage. P-values for pairwise comparisons were calculated by unpaired two-tailed Student’s t-test, *P < 0.05, **P < 0.01, ***P < 0.001, ****P < 0.0001. [file SupplementaryFile3.pdf]

Fig S4

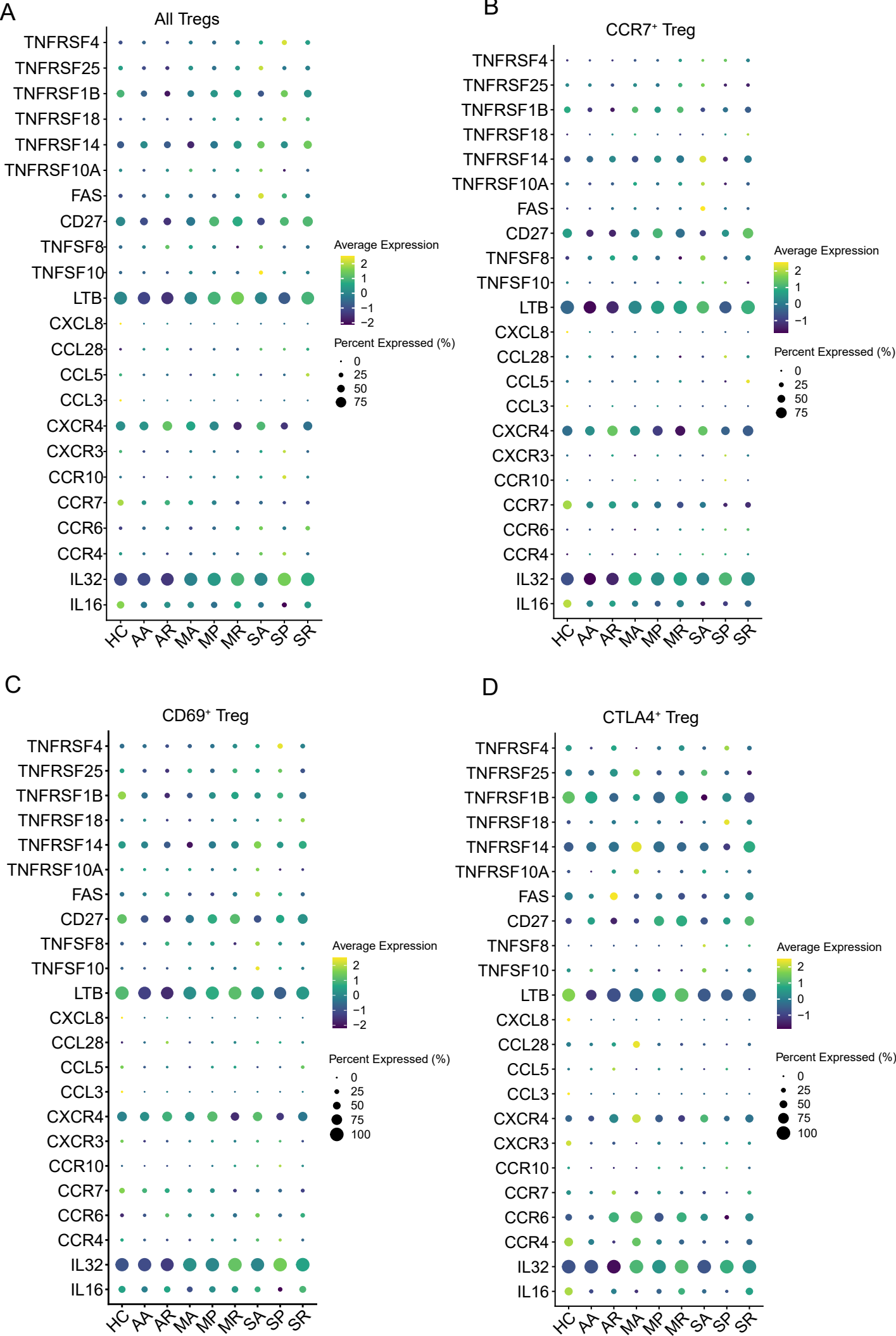

Supplement: Supplementary Figure 4 — The expression distribution of selected genes is associated with cytokine, chemokine receptor, chemokine ligand, TNF family ligand, and TNF family receptor. (A–D) The bubble heatmap plot shows the expression level of selected genes associated with cytokine, chemokine receptor, chemokine ligand, TNF family ligand, TNF family receptor in all Tregs (A), CCR7 + Tregs (B), CD69 + Tregs (C) and CTLA4 + Tregs (D). [file SupplementaryFile4.pdf]

Fig S5

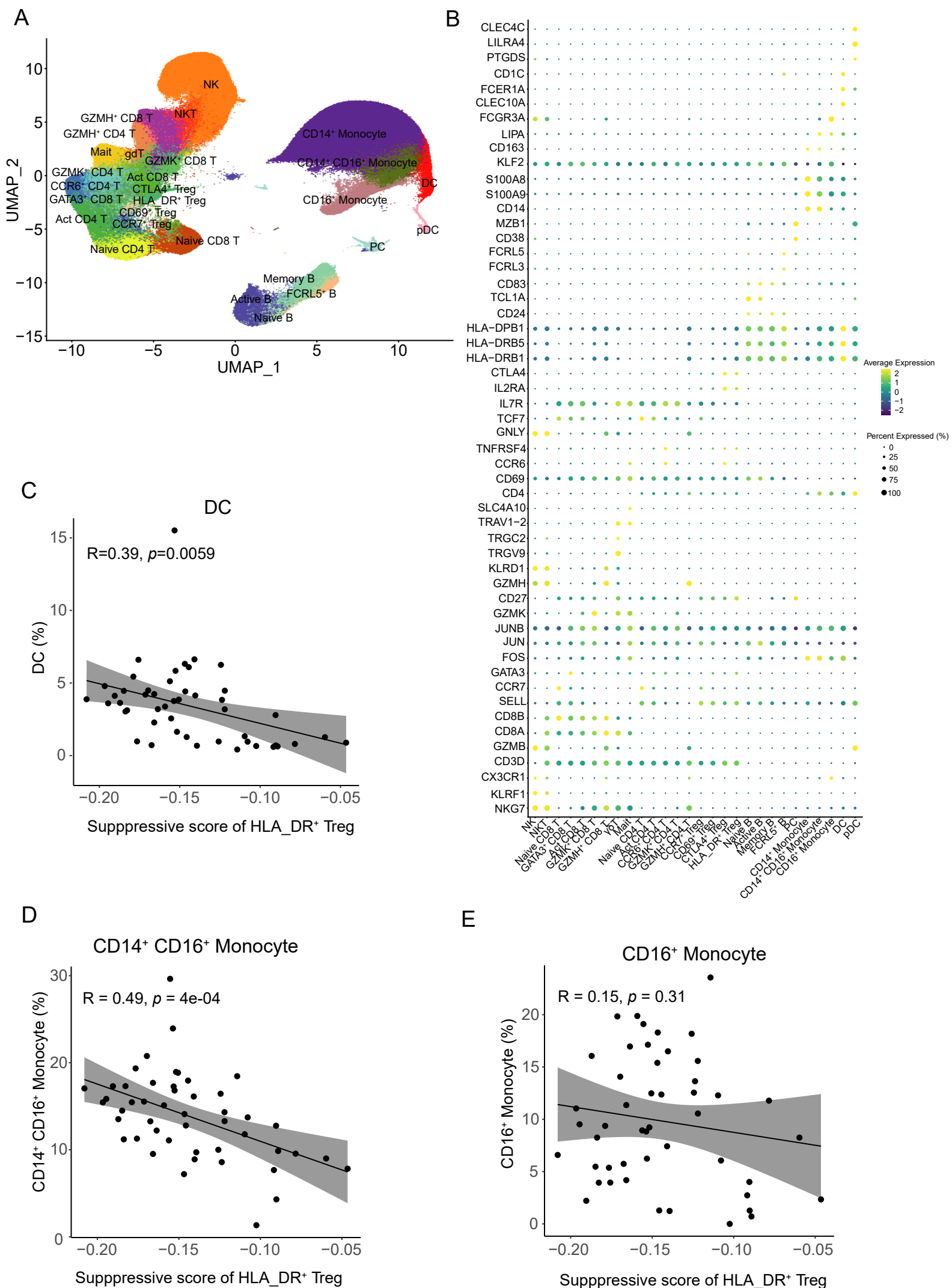

Supplement: Supplementary Figure 5 — Cellular populations of immune cells in PBMC samples from COVID-19 patients and healthy controls. (A) The UMAP plot displays the distribution of immune cells, including NK/NKT cells, T cells, B cells, myeloid cells (B) The heatmap shows the expression level of cell-specific markers for cell clusters. (C–E) The correlation between the suppressive score of HLA-DR+ Tregs and the cell percentage of DC (C), CD14+ CD16+ monocytes (D), and CD16+ monocytes (E). [file SupplementaryFile5.pdf]

Fig S6

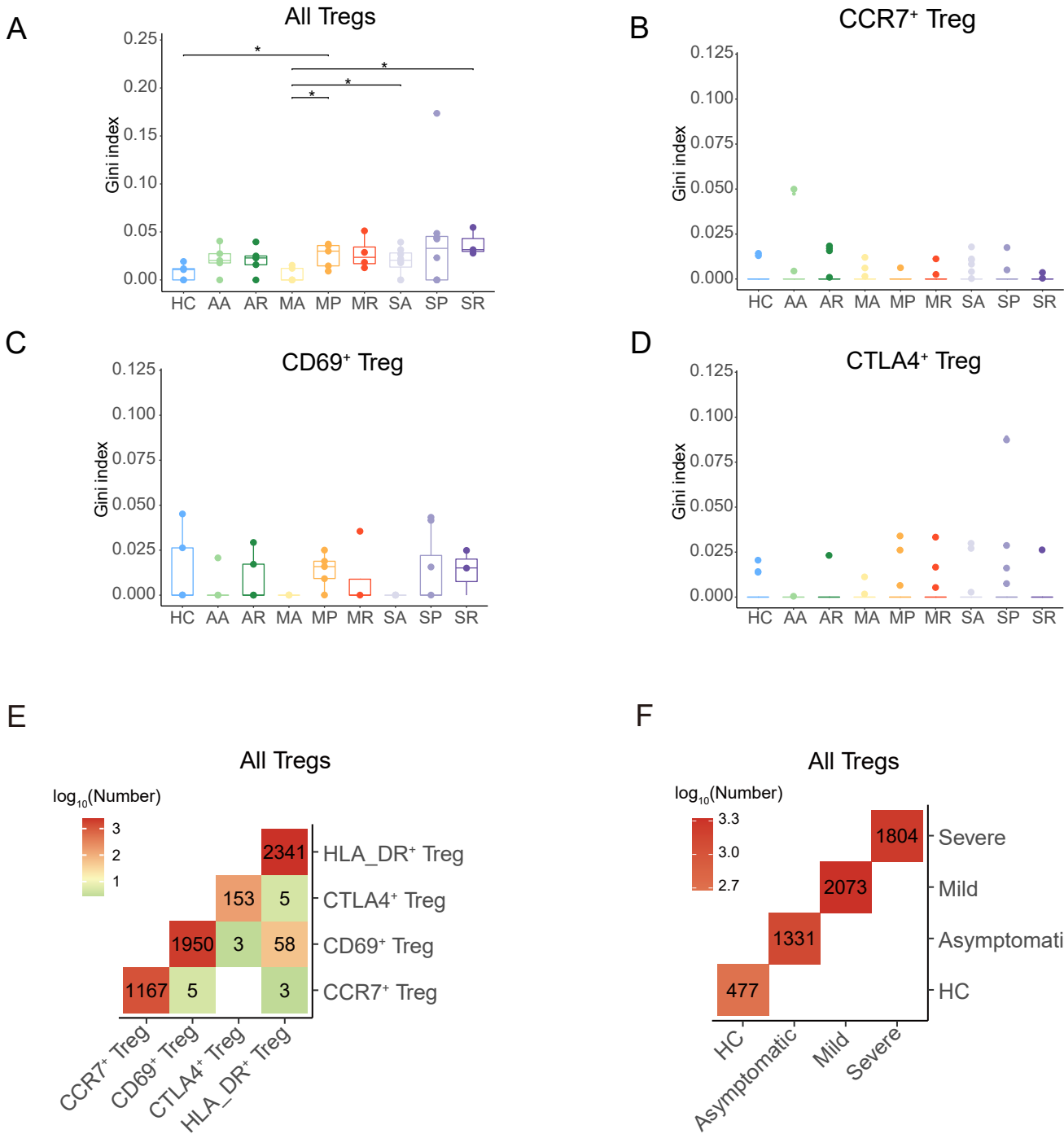

Supplement: Supplementary Figure 6 — The distribution of the Gini index in Tregs. (A–D) Distribution of the Gini index in all Tregs (A), CCR7 + Tregs (B), CD69 + Tregs (C), and CTLA4 + Tregs (D). (E, F) The sharing of TCRs among different Treg cell subpopulations (E) and across various disease stages (F). P-values for pairwise comparisons were calculated by unpaired two-tailed Student’s t-test, *P < 0.05, **P < 0.01, ***P < 0.001, ****P < 0.0001. [file SupplementaryFile6.pdf]
